# Mapping the Burden and Risk Factors of Allergic Diseases and Asthma Among Aboriginal and Torres Strait Islander People: A Scoping Review

**DOI:** 10.1111/cea.70138

**Published:** 2025-08-21

**Authors:** Desalegn Markos Shifti, Erin Pitt, Lesley Versteegh, Rani Scott Farmer, Catherine J. Hornung, Victoria Gibson, Diane Maresco‐Pennisi, Shyamali C. Dharmage, Anne B. Chang, Jennifer J. Koplin

**Affiliations:** ^1^ Child Health Research Centre The University of Queensland South Brisbane Queensland Australia; ^2^ Centre for Food Allergy Research (CFAR), Murdoch Children's Research Institute Parkville Victoria Australia; ^3^ National Allergy Centre of Excellence (NACE) Parkville Victoria Australia; ^4^ Child and Maternal Health Division and NHMRC Centre for Research Excellence in Paediatric Bronchiectasis (AusBREATHE), Menzies School of Health Research Charles Darwin University Darwin Northwest Territories Australia; ^5^ School of Nursing, Midwifery and Social Work The University of Queensland Brisbane Queensland Australia; ^6^ Centre for Clinical Research The University of Queensland Brisbane Queensland Australia; ^7^ Allergy and Lung Health Unit, School of Population and Global Health University of Melbourne Parkville Victoria Australia; ^8^ Department of Respiratory and Sleep Medicine Queensland Children's Hospital Brisbane Queensland Australia; ^9^ Australian Centre for Health Services Innovation and School of Clinical Medicine Queensland University of Technology Brisbane Queensland Australia

**Keywords:** Aboriginal and Torres Strait Islander people, allergic diseases, asthma, Australia, First Nations people, Indigenous Australians, scoping review

## Abstract

Allergic diseases and asthma are significant public health concerns in Australia and globally. However, comprehensive data on the burden among Aboriginal and Torres Strait Islander people are scarce. This scoping review aimed to systematically map existing evidence on the burden and risk factors of allergic diseases and asthma among Aboriginal and Torres Strait Islander people. MEDLINE, Scopus, Embase and Web of Science Core Collection were systematically searched through March 2024. We included studies that reported allergic diseases and asthma among Aboriginal and Torres Strait Islander people. Study characteristics and outcome data were tabulated and evidence was synthesised narratively. Fifty‐four studies involving an estimated 176,792 Aboriginal and Torres Strait Islander people were included. These studies reported on asthma (*n* = 48), eczema (*n* = 10), allergic rhinitis (*n* = 6), atopy (*n* = 3), mixed allergies (combining food, drug and other undefined allergies) (*n* = 2), and anaphylaxis (*n* = 1). No studies solely investigating food allergies were found. The majority of studies were from Western Australia (WA, *n* = 15) and the Northern Territory (NT, *n* = 14). Estimates of allergy prevalence varied widely between studies, with eczema ranging from 2.0% to 44.4%, allergic rhinitis from 0.2% to 37.3%, and atopy from 1.7% to 36.4%. Asthma prevalence ranged from 2.0% to 50.5%. Risk factors for asthma included exposure to smoke and lower socioeconomic status, while a family history of allergy was associated with an increased risk of allergic rhinitis. In conclusion, Aboriginal and Torres Strait Islander people face a potentially significant burden of allergic diseases and asthma, yet they remain underrepresented in research. Culturally responsive studies are needed to address this substantial evidence gap.

## Graphical abstract

Our study reveals Aboriginal and Torres Strait Islander people have a high allergy burden, with asthma the most prevalent; yet they remain under‐represented in research.

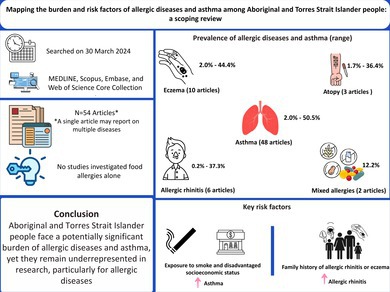

**Summary**

- In our review of allergy‐associated illness in Aboriginal and Torres Strait Islanders people, asthma was the most prevalent.
- Estimates of allergy prevalence varied widely across studies.
- Aboriginal and Torres Strait Islander people have a high allergy burden but remain under‐represented in research.

## 1 Introduction

Allergies and asthma vary widely across racial, ethnic, and socioeconomic groups around the world [1, 2, 3]. Studies [4, 5] conducted in Canada and the United States (US) revealed an increased burden of allergic and atopic conditions among Indigenous peoples in Canada. Australia currently has one of the highest prevalence rates of allergic diseases globally, with one in ten infants affected by food allergies [6], one in three children having eczema [7], nearly one in four people experiencing allergic rhinitis, one in ten having asthma and one in seven reporting other allergies [8]. However, there is a lack of comprehensive data on the burden and risk factors of allergic diseases among Aboriginal and Torres Strait Islander people.

According to the United Nations (UN), Indigenous peoples are among the most disadvantaged and vulnerable groups of people in the world today [9]. Australia's Indigenous peoples consist of many language groups, collectively called Aboriginal and Torres Strait Islander peoples [10]. Aboriginal peoples originate from all areas of mainland Australia, including Tasmania and other islands [11]. Torres Strait Islander peoples are originally from the Torres Strait Islands region between the tip of Cape York and Papua New Guinea that is made up of over two hundred islands. Aboriginal and Torres Strait Islander peoples live in urban, regional and remote areas and are present in all communities, not necessarily on their traditional lands or islands [12]. They can access primary health care in different settings, such as Aboriginal and Torres Strait Islander controlled primary health care organisations, mainstream private practices, and other public community‐based health care [13]. Despite governmental efforts to close the health gap between Aboriginal and Torres Strait Islander peoples and non‐Indigenous Australians, a substantial gap still remains. For example, the burden of disease and injury among Aboriginal and Torres Strait Islander people is 2.3 times higher compared with non‐Indigenous Australians [14].

Historically, asthma and allergies were thought by some to be uncommon in Aboriginal and Torres Strait Islander people [15]. More recent studies suggest this is not the case for asthma. In 2018–19, Aboriginal and Torres Strait Islander people were 1.6 times more likely to report having asthma compared to non‐Indigenous Australians [16]. Prevalence may vary by location. For instance, according to the National Indigenous Health Survey report, the prevalence of asthma varies from 6% in the NT to 26% in the ACT [17]. There appear to be limited studies of allergic diseases in Aboriginal and Torres Strait Islander people; however, no comprehensive scoping review of allergic diseases and asthma in these populations exists to help identify research gaps. The only available review [18] is limited to asthma and focuses solely on adults.

There has been an increasing emphasis on ‘closing the health gap’ between Aboriginal and Torres Strait Islander people and non‐Indigenous people [19, 20]. A comprehensive understanding of existing research evidence related to asthma and allergies among Aboriginal and Torres Strait Islander people may help to determine the extent to which these conditions contribute to the health burden. Our scoping review aimed to systematically map evidence on the burden and risk factors of allergic diseases and asthma among Aboriginal and Torres Strait Islander people.

## 2 Methods

This scoping review was designed and conducted according to the framework proposed by Arksey and O'Malley [21], and reported in accordance with the Preferred Reporting Items for Systematic Reviews and Meta‐Analysis extension for Scoping Reviews (PRISMA‐ScR) (Table S1) [22]. The framework consists of five steps: (1) formulating the research questions, (2) identifying relevant studies, (3) selecting eligible studies, (4) charting the data, and (5) collating, summarising and reporting the results as described below.

### 2.1 Stage 1: Formulating the Research Questions

The research questions were developed and refined through discussion and deliberation within the multidisciplinary research team. The research team includes an epidemiologist, an Indigenous researcher, a consultant respiratory physician, a specialist in allergies and chronic respiratory diseases epidemiology, and food allergy epidemiologists.

A scoping review approach was deemed appropriate given the objective and broad scope, addressing the following questions: (i) What is the burden of allergic diseases and asthma among Aboriginal and Torres Strait Islander people? and (ii) What are the risk factors associated with allergic diseases and asthma in this population?

### 2.2 Stages 2 and 3: Identifying and Selecting Relevant Studies

MEDLINE (Ovid), Scopus, Embase (Ovid) and Web of Science Core Collection were systematically searched from the inception of each database through March 2024. Google Scholar was used to identify relevant grey literature. The grey literature search also included a targeted search of documents from the latest Australian Bureau of Statistics websites that report data on the health and wellbeing of Aboriginal and Torres Strait Islander people. References of relevant adjacent reviews and included papers were screened for further relevant studies.

The search strategy included terms pertinent to allergic diseases, asthma, and Aboriginal and Torres Strait Islander people. Combinations of keywords and terms using Boolean operators, truncation, phrase searching and Medical Subject Headings (MeSH) were used in the search strategies. The search strategies and keywords used for MEDLINE were adapted for searches across all other databases (Table S1). Two authors [DMS and EP] piloted the search strategies.

Peer‐reviewed studies that reported allergic diseases and asthma among Aboriginal and Torres Strait Islander people, including prevalence, incidence, hospitalisation, time trends, risk factors and predictors/determinants, were eligible for inclusion. This encompassed clinical trials as well as cross‐sectional, case–control, cohort and qualitative studies, irrespective of study settings.

Studies were included with no limitations on the age or sex of participants. Studies were excluded if they did not provide disaggregated data on allergic diseases and/or asthma specifically for Aboriginal and Torres Strait Islander people in research involving both Aboriginal and Torres Strait Islander people and non‐Indigenous populations. Additionally, protocols, books, media articles, case reports, case studies, reviews, abstracts, commentaries and editorials were not included.

Publications were exported into EndNote 20 reference management software [23] and underwent deduplication before exportation to Covidence. Title and abstract screening and full‐text reviews were done independently by two researchers [DMS and EP] using Covidence [24]. Any discrepancies were resolved by discussion and consensus, involving a third author [JJK] when necessary.

### 2.3 Stages 4 and 5: Charting the Data, Collating, Summarising and Reporting the Results

Two reviewers [DMS and EP] piloted a data extraction template and then conducted data extraction in duplicate for all papers meeting the inclusion criteria. Any discrepancies were resolved by discussion and consensus, with the involvement of the third author [JJK] when necessary. Details extracted from each article included (i) author(s) and year of publication, (ii) study period/site/setting/inclusion/exclusion, (iii) aim(s) or research question, (iv) study design, (v) study population/sample size, (vi) data collection methods, (vii) outcome measures, (viii) key findings (demographic characteristics (age and sex), outcomes (prevalence, incidence, proportion, risk factors)) and comparison of outcomes with non‐Indigenous Australians. We consistently use the term ‘Aboriginal and Torres Strait Islander people’ to describe ‘Indigenous Australians’ and ‘First Nations people’ except when the included study specifically describes its study participants as ‘Aboriginal people' r ‘Torres Strait Islander people’ [11, 25, 26]. According to the Australian Institute of Aboriginal and Torres Strait Islander Studies (AIATSIS), the term Indigenous Australian is a broad descriptor that refers collectively to Aboriginal and Torres Strait Islander peoples [10, 11]. It should only be used when both groups are explicitly included. However, some studies in our review focus exclusively on one group, making more specific terminology necessary. For this reason, we generally use Aboriginal and Torres Strait Islander people when referring to both groups, and either Aboriginal people or Torres Strait Islander people when referring to one group specifically. These choices align with commonly accepted usage within Australia. We synthesised association data using a narrative approach. The outcomes assessed in this review encompassed various allergic diseases, including eczema, allergic rhinitis, atopy, mixed allergies, anaphylaxis and asthma as reported by the included studies (Tables S3–S9). Atopy, as reported in the included studies [27, 28], was determined through skin prick test reactions to allergens such as house dust mites, cat and dog dander, ryegrass pollen, and moulds/fungus. Mixed allergies, as described in the included study [17], involved food, drug and unspecified allergies. A forest plot was used to visually summarise the prevalence of outcomes, organised by author, year of publication, state where the study was conducted and age of participants. For studies that reported outcomes by age and/or sex rather than overall results, these specific data were incorporated into the forest plot as needed. Risk factors for allergic diseases and asthma were also examined, with effect size estimates provided where data was available.

### 2.4 Critical Appraisal

The methodological quality of the included studies was assessed using the Joanna Briggs Institute (JBI) critical appraisal tools [29, 30]. Given the inclusion of several study designs in this scoping review, multiple checklists, including those for prevalence studies, analytical cross‐sectional studies, cohort studies and quasi‐experimental studies, were used as appropriate for each study design. The quality of the included studies was categorised as ‘high’ (more than 80% ‘Yes’ responses), ‘moderate’ (50%–80% ‘Yes’ responses), or ‘low’ (less than 50% ‘Yes’ responses). Two reviewers [DMS and RSF] independently appraised the studies, with disagreements resolved through discussion.

### 2.5 Patient and Public Involvement

We acknowledge the rights of the Aboriginal and Torres Strait Islander people involved in the studies included in this review, and aim to conduct and report in accordance with the National Health and Medical Research Council (NHMRC) guidelines for reporting of health research involving Aboriginal and Torres Strait Islander people [31]. From the public involvement perspective, coauthor Mrs. Lesley Versteegh (an Arrente woman), a Research Nurse at Menzies School of Health Research, reviewed the study for its design, conduct, reporting and dissemination, in particular, the appropriateness and respect in relation to the context of Aboriginal and Torres Strait Islander people represented in this study.

### 2.6 Ethical Statement and Informed Consent

This study is a review of previously published literature and did not involve the collection of new data from human participants or animals. Therefore, ethical approval was not required. Since no personal identifiers were used and there was no direct contact with participants, informed consent was also not required.

## 3 Results

### 3.1 Study Characteristics

**FIGURE 1:**
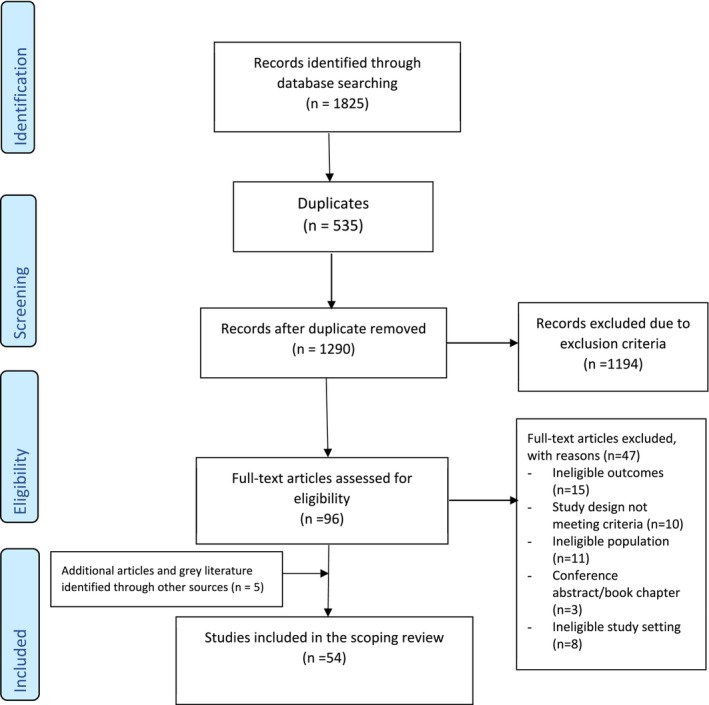
Preferred Reporting Items for Systematic Review and Meta‐analysis (PRISMA) flowchart.

The search identified 1825 papers, which were reduced to 1290 after duplicate removal. Based on the title, abstract and relevance to the research aims, 96 articles were selected for full‐text review for possible inclusion in the study. Three articles were identified through reference checks of the eligible studies, and two additional articles were found through grey literature from other sources, the Australian Bureau of Statistics. A total of 54 articles met the inclusion criteria and were included in the final analysis (Figure 1 PRISMA flowchart), involving an estimated 176,792 Aboriginal and Torres Strait Islander people with a median study sample size (interquartile range) of 538 (212–1550.5) (Tables S3–S9).

### 3.2 Quality Assessment

Only 5 studies (9.6%) [32, 33, 34, 35, 36] were rated as ‘moderate quality’, while the rest were assessed as ‘high quality’ according to the relevant JBI critical appraisal tools (Tables S11–S14).

### 3.3 Descriptive Analysis

**FIGURE 2:**
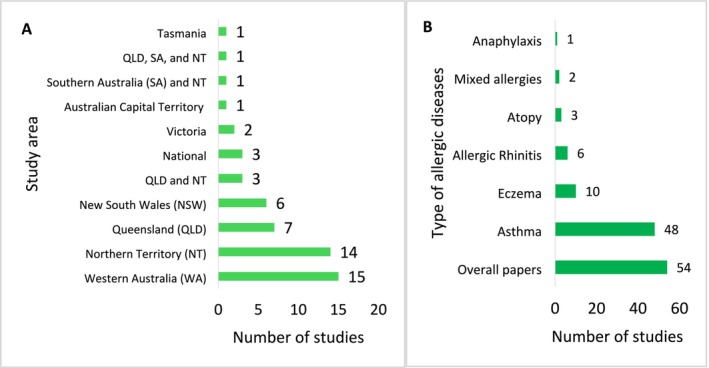
(A) The number of included publications by State/Territory and (B) the type of allergic diseases and asthma among Aboriginal and Torres Strait Islander people. ACT, Australian Capital Territory; NSW, New South Wales; NT, Northern Territory; QLD, Queensland; SA, South Australia; WA, Western Australia. For (B) one paper may report multiple allergic diseases and asthma.

**FIGURE 3:**
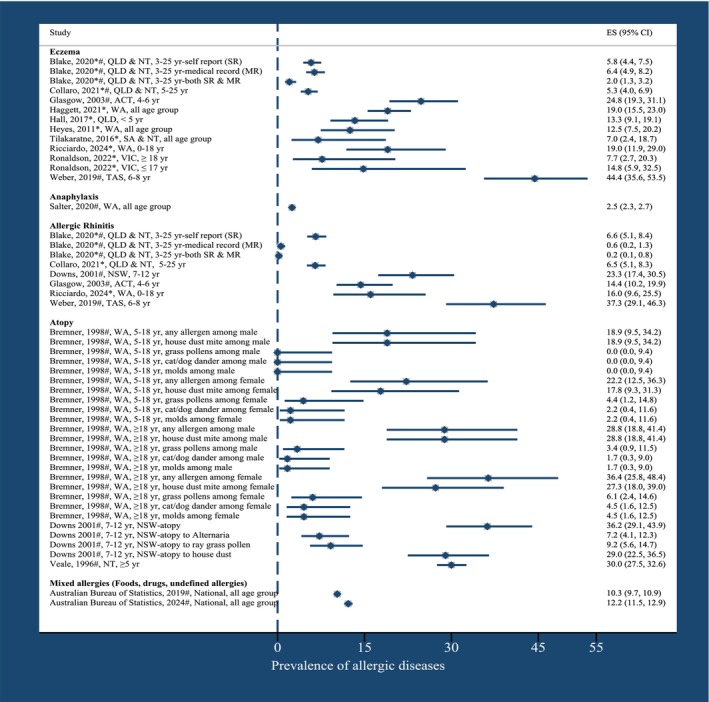
Prevalence of allergic diseases by publication year, state and age of study participants. ACT, Australian Capital Territory; NSW, New South Wales; NT, Northern Territory; QLD, Queensland; SA, South Australia; WA, Western Australia; yr., year. *Health facilities‐based studies, #Population‐based studies, *#Studies that involved both in health facilities and population‐based.

**FIGURE 4:**
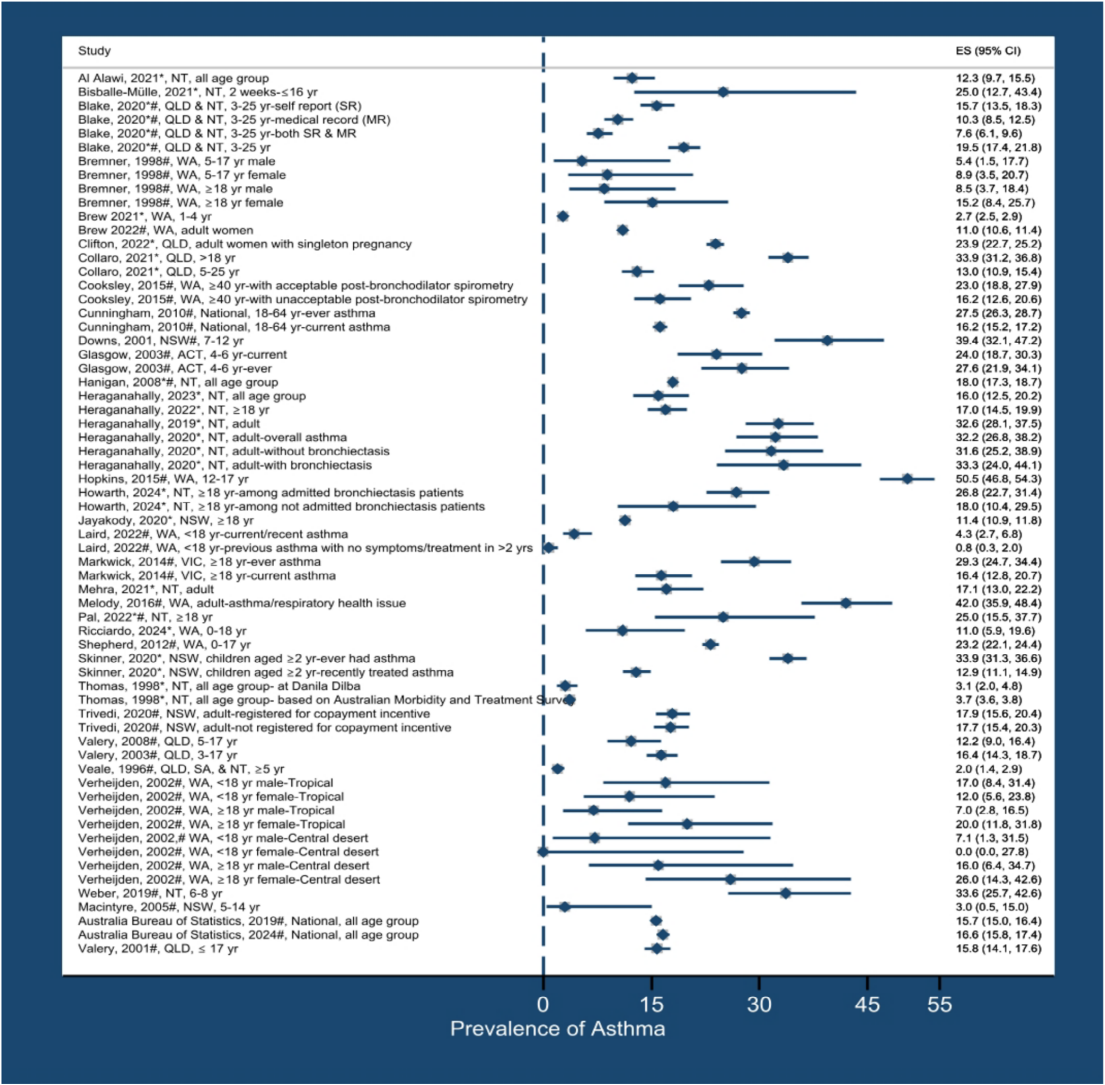
Prevalence of asthma by publication year, state/territory and age of study participants. ACT, Australian Capital Territory; NSW, New South Wales; NT, Northern Territory; QLD, Queensland; SA, South Australia; WA, Western Australia; yr., year. *Health facilities‐based studies, #Population‐based studies, *#Studies that involved both in health facilities and population‐based.

Of the 54 included studies, 43 (79.6%) were cross‐sectional [17, 28, 32, 33, 34, 35, 36, 37, 38, 39, 40, 41, 42, 43, 44, 45, 46, 47, 48, 49, 50, 51, 52, 53, 54, 55, 56, 57, 58, 59, 60, 61, 62, 63, 64, 65, 66, 67, 68, 69, 70, 71, 72, 73], 10 (18.5%) were longitudinal [71, 74, 75, 76, 77, 78, 79, 80, 81, 82], and 1 (1.9%) was a quasi‐experimental study [83]. A total of 22 studies were conducted in health facilities (hospitals, clinics and community health centres) [34, 39, 40, 41, 43, 60, 61, 62, 63, 65, 66, 67, 68, 69, 71, 75, 76, 77, 78, 79, 80, 81, 82] and 22 in population‐based settings [17, 28, 32, 35, 36, 37, 38, 47, 48, 49, 50, 51, 52, 53, 54, 55, 56, 57, 58, 59, 64, 83], with 10 involving mixed settings [33, 42, 44, 45, 46, 70, 71, 72, 73, 74] (see Figures 3 and 4 for prevalence data based on these subgroups). Asthma was the most commonly studied condition (*n* = 48) [17, 27, 32, 33, 34, 35, 36, 37, 38, 44, 45, 47, 48, 49, 50, 51, 52, 53, 54, 55, 56, 57, 58, 59, 60, 61, 62, 63, 64, 65, 66, 67, 68, 69, 70, 71, 72, 73, 74, 75, 76, 77, 78, 79, 80, 81, 82, 83, 84] followed by eczema (*n* = 10) [34, 37, 38, 39, 40, 41, 42, 43, 44, 45], allergic rhinitis/hay fever (*n* = 6) [34, 37, 38, 44, 45, 84], atopy (*n* = 3) [47, 48, 84], mixed allergies (*n* = 2) [17, 49] and anaphylaxis (*n* = 1) [46] (Figure 2B). None specifically reported on food allergy. A total of 61.1% of included studies measured allergic diseases and/or asthma as the primary outcome, with the remaining studies reporting on allergic diseases/asthma as covariates (Tables S4–S10). Studies were primarily conducted in either WA (*n* = 15) [34, 35, 36, 39, 41, 46, 47, 50, 52, 53, 55, 57, 68, 74, 77] or the NT (*n* = 14) [18, 33, 60, 61, 62, 63, 66, 67, 72, 75, 76, 79, 80, 81] (Figure 2A). Out of the 54 included studies, 22 included remote areas as part of their study [33, 34, 35, 39, 44, 45, 50, 53, 55, 56, 59, 61, 63, 64, 69, 70, 71, 74, 75, 77, 79, 80] and 17 studies did not report the specific setting (e.g., urban, rural, remote, regional or metropolitan) [32, 36, 41, 46, 47, 54, 58, 60, 66, 68, 72, 73, 76, 78, 81, 82, 83] (see Table S3). The median number of publications per year was 2 (interquartile range (IQR): 1–2.5). Publications on allergic diseases and asthma remained low over time, with only 2020–2022 exceeding three per year (see Figure S1).

### 3.4 Eczema

Figure 3 illustrates a summary of allergic diseases, including eczema, anaphylaxis, allergic rhinitis, atopy, and mixed allergies, by publication year, state and age of study participants.

Ten studies [34, 37, 38, 39, 40, 41, 42, 43, 44, 45] related to eczema were included. Eczema was assessed based on self‐report (*n* = 4) [37, 38, 40, 45], medical reviews (*n* = 2) [41, 43], physician diagnosis (*n* = 2) [39, 42], a combination of parent/self‐report and/or medical record review (*n* = 1) [44], and past medical history alongside skin examinations (*n* = 1) [34]. Three of the studies presented findings from Western Australia [34, 39, 41], two studies from QLD and NT combined [44, 45], and one study each from QLD [40], VIC [43], ACT [37], TAS [38], SA and NT combined [42] (Table S4).

The lifetime prevalence of eczema showed considerable variations, ranging from 5.8% in a study from both the NT and QLD [45] to 44.4% in TAS [38]. Studies included a range of age groups, with prevalence rates between 13.4% among children less than 5 years in QLD [40] to 44.4% among children 6–8 years old in TAS [38], 2%–11% in individuals up to 25 years old [44, 45], and 5.1%–19.0% among all age groups [39, 41, 42, 43] (Table 1). Additionally, eczema prevalence was slightly higher among Aboriginal and Torres Strait Islander people compared to non‐Indigenous Australians in two studies conducted in TAS (44.4% and 35.0%, respectively, *p* = 0.05) [38] and WA (19.0% and 17.0%, respectively) [39]; however, no statistical test results were reported for the WA study. Conversely, the prevalence of eczema was slightly lower among Aboriginal and Torres Strait Islander people compared to non‐Indigenous Australians in a single study in the ACT (25.0% and 32.0%, respectively, *p* = 0.03) [37] (Figure 3, Table S4).

**TABLE 1:** Summary of the range of prevalence estimates of allergic diseases and asthma among Aboriginal and Torres Strait Islander people based on age categories.

Allergic disease by age categories	Number of studies	Range of prevalence estimates (%)	References
Eczema			
Children 0–18 years	4	13.4–44.4	[34, 37, 38, 40]
Children and young adults (25 years)	2	2.0–11.0	[44, 45]
All ages	4	5.1–19	[39, 41, 42, 43]
Food allergies	0		
Anaphylaxis			
All ages	1	2.5	[46]
Allergic Rhinitis/Hay fever			
Children 0–18 years	4	14–37.3	[34, 37, 38, 84]
Children and young adults (25 years)	2	0.2–6	[44, 45]
Atopy			
Children 0–18 years	1	29.0	[84]
Children and adults	2	1.7–36.4	[47, 48]
Mixed allergies			
All ages	2	11.4–12.2	[17, 49]
Asthma			
Children less than 18 years	14	2.7–50.5	[32, 34, 36, 37, 38, 45, 47, 52, 56, 57, 58, 59, 64, 65, 67, 68, 76, 77, 84]
Children and young adults (25 years)	2	7.6–19.5	[44, 70]
Participants aged 5 years and above	1	2.0	[48]
Adult	16	7.0–42.0	[33, 35, 47, 50, 51, 54, 55, 57, 61, 62, 63, 66, 71, 74, 78, 80, 81, 82, 83]
All ages	4	12.4–18.0	[17, 72, 75, 79]

### 3.5 Food Allergy

No studies solely investigating food allergies were found. Only one cross‐sectional survey, which reported on food reactions among First Nations Australian children [38], was identified (Table S5). This survey, conducted among children aged 6–8 years in TAS, found that 12.9% of Aboriginal and Torres Strait Islander children had a self‐reported food reaction compared to 16.8% of non‐Indigenous children (*p* = 0.25) (Table S4).

### 3.6 Anaphylaxis

Only one study [46] reported the prevalence and trends of anaphylaxis among Aboriginal and Torres Strait Islander people and non‐Indigenous Australians across all age groups, based on a review of medical records (Figure 3). Overall, only 2.5% of anaphylaxis cases (encompassing ambulance attendance, emergency department presentations, hospital admissions and death registrations, identified through data linkage from 2002 and 2013) were Aboriginal and Torres Strait Islander people. Trends in anaphylaxis between 2002 and 2013 remained stable when comparing Aboriginal and Torres Strait Islander people with non‐Indigenous people (Figure 3, Table S6).

### 3.7 Allergic Rhinitis

Six studies that reported allergic rhinitis were included: two from multiple sites (QLD and NT) [44, 45], and one each from NSW [84], WA [34], TAS [38] and ACT [37]. Allergic rhinitis was determined based on self‐report (*n* = 3) [37, 38, 45], self‐report data and/or medical record review (*n* = 1) [44], parent‐report (*n* = 1) [84] and past medical history (*n* = 1) [34].

The prevalence of hay fever varied from 6.0% in the study conducted in both QLD and NT (among individuals aged 5–25 years) [45] to 23.3% in the study conducted in NSW (among children aged 7–12 years) [84]. Among children, the prevalence of ever having allergic rhinitis ranged between 14.0% among children aged 4–6 years in ACT to 37.3% among children aged 6–8 years in TAS [34, 37, 38, 84], while for individuals up to 25 years old, the self‐reported prevalence was 6.0% in QLD and NT combined [44, 45]. One study conducted in TAS reported allergic rhinitis (37.3%) and hay fever (22.6%) separately [38].

The prevalence of hay fever was lower among Aboriginal and Torres Strait Islander people compared with non‐Indigenous Australians in two studies. In the ACT, the prevalence was 14% for Aboriginal and Torres Strait Islander people and 19% for non‐Indigenous people [37]. In NSW, the prevalence is 23.3% for Aboriginal and Torres Strait Islander people and 35.2% for non‐Indigenous people (*p <* 0.05) [84] (Figure 3, Table S7).

The study by Downs et al. [84] was the only paper we reviewed that identified risk factors for hay fever among Aboriginal and Torres Strait Islander people. Having a parent with hay fever or eczema (AOR = 17.9, 95% CI: 3.5–90.8) increased the odds of hay fever (Table S7).

### 3.8 Atopy

Three studies [47, 48, 84] that reported atopy based on the skin prick test (SPT) were included (Table S5). The prevalence of atopy ranged from 1.7% (due to cat/dog dander and moulds among adult males aged 18 and older) in WA in 1998 [47] to 36.2% (due to Alternaria among children aged 7–12) in NSW in 2001 [28]. Bremner et al.'s 1998 [47] study conducted in WA showed that atopy was more common in adults than in children, among non‐Indigenous people than Aboriginal and Torres Strait Islander people, and in women than in men. For instance, Bremner et al. [47] reported that among Aboriginal and Torres Strait Islander people aged 18 years and above, the prevalence of atopy in males and females, respectively, was 28.8% and 36.4% for any allergens, 28.8% and 27.3% for house dust mites, 3.4% and 6.1% for grass pollen, 1.7% and 4.5% for cat/dog dander, and 1.7% and 4.5% for moulds (Figure 3). Among non‐Indigenous Australians aged 18 years and above, the prevalence of atopy in males and females, respectively, was 53.1% and 52.4% for any allergens, 43.3% and 42.9% for house dust mites, 33.2% and 36.0% for grass pollen, 25.3% and 36.0% for cat/dog dander, and 19.1% and 16.0% for moulds (Figure 3, Table S8).

### 3.9 Mixed Allergies

Two national surveys [17, 49] reported the prevalence of self‐reported mixed allergies, defined as food, drug and unspecified allergies. The prevalence of mixed allergies was 12.2% (14.7% among males and 9.5% among females) in 2022–23 [49]. The prevalence of age‐standardised mixed allergies in 2018–19 was 11.4% among Aboriginal and Torres Strait Islander people, compared to 12.2% among non‐Indigenous Australians [17]. This survey also reported that after adjusting for sex, the prevalence of these allergies among Aboriginal and Torres Strait Islander people was 13.7% among females compared to 8.7% among males (Figure 3, Table S9).

### 3.10 Asthma

Figure 4 presents a summary of asthma by publication year, state and age of study participants.

A total of 48 articles related to asthma were identified, with 13 studies conducted in NT [33, 60, 61, 62, 63, 66, 67, 72, 75, 76, 79, 80, 81], 12 in WA [34, 35, 36, 47, 50, 52, 53, 55, 57, 68, 74, 77], 6 in QLD [32, 56, 59, 64, 71, 78] and NSW [58, 65, 73, 82, 83, 84] each, 3 in both QLD and NT [44, 70, 71] and nationwide [17, 49, 51] and 1 each in ACT [37], TAS [38], VIC [54], multiple cities (QLD, SA and NT) [48] and Central Australia [69]. Asthma was identified using medical record review (*n* = 19) [33, 34, 55, 61, 62, 63, 64, 66, 67, 68, 69, 71, 72, 73, 77, 78, 79, 81, 82], self‐report (*n* = 10) [17, 27, 32, 35, 37, 49, 50, 52, 56, 83], parent/primary carer report (*n* = 5) [36, 53, 58, 65, 84], self‐reported doctor or nurse diagnosis (*n* = 4) [38, 51, 54, 57], standard clinical criteria (*n* = 4) [48, 56, 76, 80], self‐report and/or medical record review (*n* = 4) [44, 45, 60, 74], and not reported (*n* = 1) [75] (Table S10).

The prevalence of asthma ranged from 2.0% in the multi‐site study involving rural communities in QLD, SA and NT in 1990–91 [48] to 50.5% among the urban population of WA in 2000–02 [52]. A national survey in 2018–19 reported an overall prevalence of 15.7%, with a higher rate of 17.3% in non‐remote areas compared to 8.6% in remote areas [17]. Among studies involving children aged less than 18, the prevalence varied substantially, from 2.7% to 50.5% [32, 34, 36, 37, 38, 45, 47, 52, 56, 57, 58, 64, 65, 67, 68, 76, 77, 84], while across all age groups, it ranged between 12.4% and 18.0% [17, 72, 75, 79] (Table 1).

Eighteen studies [33, 37, 38, 47, 50, 51, 54, 56, 58, 63, 71, 72, 75, 76, 78, 80, 82, 84] compared the prevalence of asthma among Aboriginal and Torres Strait Islander people and non‐Indigenous Australians. *p*‐values or 95% CI are reported only when they are provided in the studies. Glasgow et al. [37] reported current asthma prevalence rates of 24% among Aboriginal and Torres Strait Islander children aged 4–6 years in the ACT, compared to 15% among non‐First Nations children of the same age group. Similarly, Cunningham et al. [51] reported national current asthma prevalence rates of 16.2% (95% CI 14.6–17.8) among Aboriginal and Torres Strait Islander adults aged 18–64 years, compared to 9.9% (95% CI 9.3–10.4) among non‐First Nations adults in the same age group. In contrast, Bisballe‐Mülle et al. [76] reported an asthma prevalence of 25.0% among hospitalised Aboriginal and Torres Strait Islander children aged 2 weeks to ≤ 16 years in NSW, compared to 75.0% among non‐First Nations children in the same age group. Collaro et al. [71] reported a comparable asthma prevalence rate of 34.0% among Aboriginal and Torres Strait Islander adults aged > 18 years in QLD and 30.0% among non‐First Nations adults of the same age (Figure 4, Table 1, Table S10).

Thirty‐seven articles reported on the prevalence of asthma [33, 34, 35, 36, 37, 38, 44, 45, 47, 48, 50, 51, 52, 53, 54, 56, 57, 58, 59, 62, 63, 64, 65, 66, 70, 71, 74, 75, 76, 77, 78, 79, 80, 81, 82, 83, 84], 14 articles on risk factors for asthma [35, 36, 45, 48, 51, 52, 53, 60, 69, 73, 77, 78, 80, 84], five articles on admission to health facilities [60, 67, 68, 72, 77], one article on the trend in asthma‐related hospital admissions over time [55], and one on risk factors for first asthma hospitalisation [73] (Table S10).

Factors increasing the odds of asthma among children included ever having pneumonia (OR = 3.10, 95% CI: 1.49, 6.45), pneumonia either before age three (OR = 2.67, 95% CI 1.04–6.85) or between three and 5 years of age (OR = 8.23, 95% CI: 2.73, 24.8) [45], as well as atopy and parent history of asthma, hay fever or eczema [84]. Children whose families reported running out of food and being unable to afford to buy more food in the last 12 months (AOR = 1.3, 95% CI: 1.01, 1.70) [51], and those in the top quantile of the Index of Relative Indigenous Socioeconomic Outcomes (indicating greater economic disadvantage) (OR = 9.2, 95% CI: 3.1, 27.2) [36] were also at a higher risk of having asthma. Environmental risk factors for asthma included exposure to prenatal maternal smoking (posterior probability (PR) = 1.18, 95% credible interval (CrI): 1.00, 1.40) [65] and allergy to cats (AOR = 2.5, 95% CI: 1.3, 5.0) [48], among others. Risk factors associated with hospital admission due to asthma were being hospitalised for an acute respiratory tract infection (OR = 4.06, 95% CI: 3.44, 4.78), living in a disadvantaged area (OR = 1.58, 95% CI: 1.28, 1.94), being born at less than 33 weeks of gestation (OR = 3.30, 95% CI: 2.52, 4.32) and birth weight < 1500 g (OR = 2.35, 95% CI: 1.39, 3.99) [77] (Table S10).

A few studies have identified factors that reduce the risk of asthma. There were lower odds of self‐reported asthma among Aboriginal and Torres Strait Islander people with non‐English as a main language (AOR = 0.5, 95% CI: 0.3, 0.8) and remote and very remote areas of residence (AOR = 0.5, 95% CI = 0.3, 0.07) [51]. In a study conducted over two decades ago (1999) [67], Wyhbourne et al. reported that the risk ratio for Aboriginal and Torres Strait Islander children hospitalised with asthma as a principal diagnosis, compared to non‐Indigenous Australian children, was 0.32 (95% CI: 0.26, 0.40) (Table S10).

## 4 Discussion

To our knowledge, our study is the first comprehensive scoping review mapping the evidence on the burden and risk factors of allergic diseases and asthma among Aboriginal and Torres Strait Islander people. Of the 54 studies identified, most were conducted in WA (*n* = 15) and NT (*n* = 14), focusing mainly on asthma (*n* = 48), followed by eczema (*n* = 10) and allergic rhinitis (*n* = 6). A notable evidence gap was identified for food allergy, with no studies investigating this outcome despite its potential severity and co‐occurrence with other allergic diseases [6, 85].

Prevalence estimates varied widely between studies. For asthma, prevalence among Aboriginal and Torres Strait Islander people varied from 2.0% in a study conducted across combined rural areas of QLD, SA and the NT among children aged 5 and older (1996) [48], to 50.0% among 12–17‐year‐olds in an urban area of WA (2015) [52]. Despite both studies relying on self/parent‐reported data [52], prevalence estimates for eczema ranged from 5.0% in the NT and QLD combined (2021) [45] to 44.4% in TAS (2019) [38], while allergic rhinitis prevalence estimates varied from 6.0% in QLD and the NT combined (2021) [45] to 23.3% in NSW (2001) [84]. These findings highlight a potential variation in allergic diseases and asthma prevalence across Australia's states and territories, underscoring the need for targeted region‐specific research. Other factors that may have contributed to the observed variations in the prevalence of allergic diseases and asthma between studies include differences in outcome measurement (e.g., self‐reported data versus medical record review), study populations (e.g., clinical cohorts versus population‐based samples), methodologies (e.g., cultural appropriateness, involvement of local Elders, and the use of local languages) and study quality (e.g., sample size and response rates). Additional area‐specific risk factors, such as age distribution, remoteness, healthcare access, housing conditions and smoking rates, may also contribute. For instance, approximately 30% of Aboriginal and Torres Strait Islander people reported unmet healthcare needs in the 12 months preceding the 2018–19 National Indigenous Health Survey [86]. This is a concern because 22 of the 54 included studies were conducted in health facilities, likely excluding individuals who visit these facilities infrequently, as well as potentially missing milder or undiagnosed cases. While health facility‐based studies are relatively cost‐effective and yield outcomes diagnosed by health professionals, they may not represent the general population comprehensively. Additionally, only 61% of the included studies specifically measured allergic diseases and/or asthma as outcomes, potentially leading to under‐ or over‐estimation of prevalence in studies where these conditions were not the primary focus.

Studies comparing asthma prevalence between Aboriginal and Torres Strait Islander people and non‐Indigenous Australians also reveal notable variations based on state/territory, study settings and age groups. Glasgow et al. [37] and Cunningham et al. [51] reported higher asthma prevalence rates among Aboriginal and Torres Strait Islander children (24% in the ACT) and adults (16.2% nationally) compared to non‐Indigenous Australians in similar age groups (15% and 9.9%, respectively). While the current review synthesised evidence available until 2024, a recently published study [87] further supports these findings, demonstrating that Aboriginal and Torres Strait Islander people in Central Queensland were significantly more likely to present to emergency departments for asthma and other allergic diseases compared to non‐Indigenous Australians. Conversely, Bisballe‐Mülle et al. [76] found lower asthma prevalence among hospitalised Aboriginal and Torres Strait Islander children (25%) compared to non‐Indigenous children (75%) in NSW. Collaro et al. [71], however, reported comparable prevalence rates among Aboriginal and Torres Strait Islander and non‐First Nations adults in QLD (34% versus 30%). These findings highlight the need for tailored, context‐specific interventions to address higher prevalence rates, potential underdiagnosis in certain states or territories, and disparities in healthcare utilisation.

Key drivers of asthma among Aboriginal and Torres Strait Islander people identified from the included studies include disadvantaged socioeconomic status [51], exposure to smoking [65], pneumonia among children [45], atopy, parent history of asthma, hay fever or eczema [84] and allergy to pets [48] among other factors. The Australian Burden of Disease Study reported that almost half (47%) of the respiratory disease burden among Indigenous Australians in 2018 was attributed to smoking [88]. These findings are consistent with an international study among Indigenous adolescents in Canada that identified income, education and smoking inside the home as significant factors associated with asthma [89].

No studies have exclusively reported food allergies among Aboriginal and Torres Strait Islander people, except for a national survey [17] which reported 11.4% prevalence of mixed allergies (food, drug and undefined allergies combined), compared to 12.2% among non‐Indigenous Australians. The lack of literature on food allergy among Aboriginal and Torres Strait Islander people is concerning, particularly given Australia's status as having the highest global prevalence of food allergies, affecting 11.3% of infants and 6.3% of children up to the age of 10 years [6, 85, 90, 91]. Existing studies [6, 90, 92, 93, 94] on food allergies in Australia have predominantly focused on urban populations and lack data based on Indigenous status. In contrast, a nationwide study among Indigenous people in Canada described a 4.9% prevalence of self‐reported food allergies, compared to 8.1% among the general population [95]. There is an urgent need for culturally responsive research to better understand and monitor the burden and impacts of food allergies among Aboriginal and Torres Strait Islander people.

Eczema was the second most commonly investigated condition after asthma. Eczema is generally more common in children than adults [7] and prevalence rates in children in the included studies ranged between 13.4% among children aged less than 5 years to 44.4% among children aged 6–8 years [38, 40]. Eczema is a known risk factor for other atopic disorders, such as asthma, hay fever, food allergy and eosinophilic esophagitis [96]. It also increases susceptibility to streptococcal skin infections [96, 97, 98], and if left untreated, can lead to severe, potentially life‐threatening complications, including sepsis, endocarditis and bone and joint infections [96, 99, 100, 101]. Findings regarding eczema prevalence in Aboriginal and Torres Strait Islander children compared to their non‐Indigenous counterparts were mixed, with higher rates in a study in TAS (44.4% vs. 35.0%) [38], lower rates in a study in the ACT (25% vs. 32%) [37] and similar rates in WA (19.0% vs. 17.0%) [39]. A systematic review of urban‐living Indigenous children and young people (CYP) in high‐income countries found that current and severe symptoms of eczema were more common among urban‐living Indigenous CYP compared to their non‐Indigenous peers, with children showing a higher prevalence than adolescents [102]. Further research is needed to explore the reasons for these inconsistent findings, including whether geographic variations contribute to the differences between studies.

Our scoping review identified one study [46] reporting a cohort of people experiencing anaphylaxis between 2002 and 2013, in which Aboriginal and Torres Strait Islander people accounted for 2.5% of cases. Most existing Australian studies on anaphylaxis [103, 104, 105, 106, 107] lack data disaggregated by Indigenous status, obscuring the full extent of any disparities. This highlights a critical gap in understanding the burden of this acute and potentially life‐threatening condition among Aboriginal and Torres Strait Islander populations.

The majority of research was based in WA (*N* = 15) and the NT (*N* = 14). However, there is a substantial gap in research in other states with a high proportion of Aboriginal and Torres Strait Islander people, such as NSW and QLD. NSW has the largest proportion of Aboriginal and Torres Strait Islander people, accounting for 34.5% of the total Aboriginal and Torres Strait Islander population, followed by QLD with 27.8% [108]. Overall, Aboriginal and Torres Strait Islander people represent approximately 3.8% of Australia's population [109]. They are not one group but comprise hundreds of groups that have their own distinct set of languages, histories and cultural traditions [110]. Therefore, the prevalence and risk factors of allergic diseases and asthma could vary across different contexts. Our findings highlight the inequitable distribution of research on Aboriginal and Torres Strait Islander people across states and geographic regions. Most importantly, it underscores the significant gap in evidence regarding context‐specific burden and risk factors of allergic diseases and asthma among Aboriginal and Torres Strait Islander people. Collaboration at the local, state/territory, and national levels is crucial to bridging the gap in research on allergic diseases and asthma, ensuring that the burden and context‐specific risk factors affecting Aboriginal and Torres Strait Islander people are addressed.

Among the 54 included studies, 22 included remote areas, three were national surveys (two independent national surveys and one derived from national survey data), and 17 did not report their study settings (e.g., urban, rural, remote, regional or metropolitan). Additionally, most studies did not report outcomes by specific contexts, such as urban, rural, remote, regional or metropolitan areas, beyond merely describing them as study settings. This gap hinders our understanding of the context‐specific prevalence, rates and risk factors of allergic diseases and asthma. Studies in other countries highlight disparities in allergic disease prevalence between rural and urban areas. For instance, a study in South Africa found a higher rate of both self‐reported and objectively measured food allergies in urban regions compared to rural ones, and a lower prevalence in a rural black African cohort compared with urban black African participants [111]. The lack of specificity in existing Australian studies impedes the effective application of targeted intervention through precision public health [112], which aims to deliver ‘the right intervention at the right time, every time, to the right population’.

Overall, our study reveals a striking paucity of research on allergic diseases among Aboriginal and Torres Strait Islander people, despite Australia being renowned as the ‘allergy capital of the world’ [113]. This gap persists over time, with only the period from 2020 to 2022 surpassing three studies per year, underscoring the urgent need for increased focus in this area. There is a pressing need for additional research to address the substantial evidence gap regarding allergic diseases among Aboriginal and Torres Strait Islander people. Such studies are essential for understanding the current burden, tracking changes over time, predicting future trends and implementing evidence‐based, targeted interventions. While obtaining data is crucial, it is equally essential that studies involving Indigenous people are undertaken within an Indigenous framework [114]. This means research questions, methods and data should be co‐designed in collaboration and equal partnership with the local Indigenous community [115, 116]. Although our scoping review did not specifically extract this data, it was often not clearly described in the included articles, thus, this remains an area for further improvement.

A key strength of our study is the systematic approach to identifying relevant literature to map the evidence on the burden and risk factors of allergic diseases and asthma among Aboriginal and Torres Strait Islander people, drawing from both published and grey literature. It includes articles from the inception of each database up to the most recent updates. A limitation is the availability of the data whereby there was marked heterogeneity in study designs and settings across the included studies, which limits the comparability of the included studies and the ability to draw overall conclusions about prevalence and risk factors.

## 5 Conclusion

This review highlighted that Aboriginal and Torres Strait Islander people face a potentially significant burden of allergic diseases and asthma, yet they remain underrepresented in research, particularly for allergic diseases. As a result, little is known about the prevalence and risk factors of common allergic diseases among Aboriginal and Torres Strait Islander people. There appear to be substantial differences in the burden of allergic diseases and asthma across states/territories of Australia and between remote and non‐remote areas; however, further studies are needed to confirm this, particularly for allergic diseases. Culturally responsive studies are urgently needed to understand and address these important evidence gaps.

## Author Contributions

All authors, D.M.S., E.P., L.V., R.S.F., C.J.H., V.G., D.M.‐P, S.C.D., A.B.C., J.J.K., conceived the idea and designed the methodology. D.M.S. and E.P. performed title and abstract screening, full‐text review, and data extraction. D.M.S. and R.S.F. conducted the quality assessment. D.M.S. analysed the data and drafted the manuscript. All authors critically reviewed and approved the manuscript.

## Conflicts of Interest

S.C.D. has received independent investigator‐initiated grants from G.S.K., AstraZeneca and Sanofi for unrelated research. J.J.K. was a named investigator on a grant from Sanofi Regeneron for unrelated research. J.J.K. received a research award from the Stallergenes Greer Foundation, paid to her institution, unrelated to the current manuscript. A.C. declares multiple grants to her institutions from the Australian National Health Medical Research Council (NHMRC) and Medical Research Future Funds (MRFF) related to studies on children's Indigenous lung health, chronic cough and bronchiectasis. A.C. receives funds to her institution for being an independent data management committee member for clinical trials of unlicensed vaccines (GlaxoSmithKline and Moderna), and monoclonal antibody (AstraZeneca); an advisory member of study design for unlicensed products for bronchiectasis (Boehringer‐Ingelheim and Zambon), advisory diversity committee member (Boehringer‐Ingelheim) and personal fees from being an author of two UpToDate chapters that are outside the submitted work. All remaining authors declare no conflicts of interest.

## Supporting information


**Appendix S1:** cea70138‐sup‐0001‐AppendixS1.docx.

## Data Availability

The authors have nothing to report.
