# Supplementary material for: Mapping the Burden and Risk Factors of Allergic Diseases and Asthma Among Aboriginal and Torres Strait Islander People: A Scoping Review
Source: Clin Exp Allergy. 2025 Aug 21;56(1):15–29. doi: 10.1111/cea.70138 (PMC12774580; doi:10.1111/cea.70138)
Supplement: Supplementary file 1 — Appendix S1: cea70138‐sup‐0001‐AppendixS1.docx. [file CEA-56-15-s001.docx]

**Supplemental Table 1.** Preferred Reporting Items for Systematic reviews and Meta-Analyses extension for Scoping Reviews (PRISMA-ScR) Checklist

| **SECTION** | **ITEM** | **PRISMA-ScR CHECKLIST ITEM** | **REPORTED ON PAGE #** |
| --- | --- | --- | --- |
| **TITLE** | | | |
| Title | 1 | Identify the report as a scoping review. | 1 |
| **ABSTRACT** | | | |
| Structured summary | 2 | Provide a structured summary that includes (as applicable): background, objectives, eligibility criteria, sources of evidence, charting methods, results, and conclusions that relate to the review questions and objectives. | 3 |
| **INTRODUCTION** | | | |
| Rationale | 3 | Describe the rationale for the review in the context of what is already known. Explain why the review questions/objectives lend themselves to a scoping review approach. | 4 and 5 |
| Objectives | 4 | Provide an explicit statement of the questions and objectives being addressed with reference to their key elements (e.g., population or participants, concepts, and context) or other relevant key elements used to conceptualize the review questions and/or objectives. | 5 |
| **METHODS** | | | |
| Protocol and registration | 5 | Indicate whether a review protocol exists; state if and where it can be accessed (e.g., a Web address); and if available, provide registration information, including the registration number. | N/A |
| Eligibility criteria | 6 | Specify characteristics of the sources of evidence used as eligibility criteria (e.g., years considered, language, and publication status), and provide a rationale. | 5 and 6 |
| Information sources* | 7 | Describe all information sources in the search (e.g., databases with dates of coverage and contact with authors to identify additional sources), as well as the date the most recent search was executed. | 5 and 6 |
| Search | 8 | Present the full electronic search strategy for at least 1 database, including any limits used, such that it could be repeated. | 6, 40-45 |
| Selection of sources of evidence† | 9 | State the process for selecting sources of evidence (i.e., screening and eligibility) included in the scoping review. | 5 and 6 |
| Data charting process‡ | 10 | Describe the methods of charting data from the included sources of evidence (e.g., calibrated forms or forms that have been tested by the team before their use, and whether data charting was done independently or in duplicate) and any processes for obtaining and confirming data from investigators. | 6 and 7 |
| Data items | 11 | List and define all variables for which data were sought and any assumptions and simplifications made. | 7 |
| Critical appraisal of individual sources of evidence§ | 12 | If done, provide a rationale for conducting a critical appraisal of included sources of evidence; describe the methods used and how this information was used in any data synthesis (if appropriate). | 7 and 8 |
| Synthesis of results | 13 | Describe the methods of handling and summarizing the data that were charted. | 6 and 7 |
| **RESULTS** | | | |
| Selection of sources of evidence | 14 | Give numbers of sources of evidence screened, assessed for eligibility, and included in the review, with reasons for exclusions at each stage, ideally using a flow diagram. | 8 and 9 |
| Characteristics of sources of evidence | 15 | For each source of evidence, present characteristics for which data were charted and provide the citations. | 10 |
| Critical appraisal within sources of evidence | 16 | If done, present data on critical appraisal of included sources of evidence (see item 12). | 9 |
| Results of individual sources of evidence | 17 | For each included source of evidence, present the relevant data that were charted that relate to the review questions and objectives. | 11-18 |
| Synthesis of results | 18 | Summarize and/or present the charting results as they relate to the review questions and objectives. | 12, 15, and 18 |
| **DISCUSSION** | | | |
| Summary of evidence | 19 | Summarize the main results (including an overview of concepts, themes, and types of evidence available), link to the review questions and objectives, and consider the relevance to key groups. | 18 and 23 |
| Limitations | 20 | Discuss the limitations of the scoping review process. | 23 |
| Conclusions | 21 | Provide a general interpretation of the results with respect to the review questions and objectives, as well as potential implications and/or next steps. | 23 and 24 |
| **FUNDING** | | | |
| Funding | 22 | Describe sources of funding for the included sources of evidence, as well as sources of funding for the scoping review. Describe the role of the funders of the scoping review. | 2, 48-85 |

JBI = Joanna Briggs Institute; PRISMA-ScR = Preferred Reporting Items for Systematic reviews and Meta-Analyses extension for Scoping Reviews.

* Where *sources of evidence* (see second footnote) are compiled from, such as bibliographic databases, social media platforms, and Web sites.

† A more inclusive/heterogeneous term used to account for the different types of evidence or data sources (e.g., quantitative and/or qualitative research, expert opinion, and policy documents) that may be eligible in a scoping review as opposed to only studies. This is not to be confused with *information sources* (see first footnote).

‡ The frameworks by Arksey and O’Malley (6) and Levac and colleagues (7) and the JBI guidance (4, 5) refer to the process of data extraction in a scoping review as data charting*.*

§ The process of systematically examining research evidence to assess its validity, results, and relevance before using it to inform a decision. This term is used for items 12 and 19 instead of "risk of bias" (which is more applicable to systematic reviews of interventions) to include and acknowledge the various sources of evidence that may be used in a scoping review (e.g., quantitative and/or qualitative research, expert opinion, and policy document).

**Supplemental Table** **2.** MEDLINE search strategies and results

| # | Query | Results |
| --- | --- | --- |
| 1 | (food? adj3 sensiti$).mp. [mp=title, book title, abstract, original title, name of substance word, subject heading word, floating sub-heading word, keyword heading word, organism supplementary concept word, protocol supplementary concept word, rare disease supplementary concept word, unique identifier, synonyms, population supplementary concept word, anatomy supplementary concept word] | 3,108 |
| 2 | (food? adj3 toleran$).mp. [mp=title, book title, abstract, original title, name of substance word, subject heading word, floating sub-heading word, keyword heading word, organism supplementary concept word, protocol supplementary concept word, rare disease supplementary concept word, unique identifier, synonyms, population supplementary concept word, anatomy supplementary concept word] | 1,141 |
| 3 | (food? adj3 intoleran$).mp. [mp=title, book title, abstract, original title, name of substance word, subject heading word, floating sub-heading word, keyword heading word, organism supplementary concept word, protocol supplementary concept word, rare disease supplementary concept word, unique identifier, synonyms, population supplementary concept word, anatomy supplementary concept word] | 1,787 |
| 4 | allerg$.mp. [mp=title, book title, abstract, original title, name of substance word, subject heading word, floating sub-heading word, keyword heading word, organism supplementary concept word, protocol supplementary concept word, rare disease supplementary concept word, unique identifier, synonyms, population supplementary concept word, anatomy supplementary concept word] | 261,602 |
| 5 | ((aero or air$) adj3 allergen?).mp. [mp=title, book title, abstract, original title, name of substance word, subject heading word, floating sub-heading word, keyword heading word, organism supplementary concept word, protocol supplementary concept word, rare disease supplementary concept word, unique identifier, synonyms, population supplementary concept word, anatomy supplementary concept word] | 3,207 |
| 6 | (aeroallergen? adj3 sensiti$).mp. [mp=title, book title, abstract, original title, name of substance word, subject heading word, floating sub-heading word, keyword heading word, organism supplementary concept word, protocol supplementary concept word, rare disease supplementary concept word, unique identifier, synonyms, population supplementary concept word, anatomy supplementary concept word] | 918 |
| 7 | (allergen? adj3 sensiti$).mp. [mp=title, book title, abstract, original title, name of substance word, subject heading word, floating sub-heading word, keyword heading word, organism supplementary concept word, protocol supplementary concept word, rare disease supplementary concept word, unique identifier, synonyms, population supplementary concept word, anatomy supplementary concept word] | 5,632 |
| 8 | Hypersensitivity, Immediate/ | 13,160 |
| 9 | hypersensitiv$.mp. [mp=title, book title, abstract, original title, name of substance word, subject heading word, floating sub-heading word, keyword heading word, organism supplementary concept word, protocol supplementary concept word, rare disease supplementary concept word, unique identifier, synonyms, population supplementary concept word, anatomy supplementary concept word] | 202,060 |
| 10 | Hypersensitivity/ | 53,562 |
| 11 | exp Food Hypersensitivity/ | 25,303 |
| 12 | asthma$.mp. [mp=title, book title, abstract, original title, name of substance word, subject heading word, floating sub-heading word, keyword heading word, organism supplementary concept word, protocol supplementary concept word, rare disease supplementary concept word, unique identifier, synonyms, population supplementary concept word, anatomy supplementary concept word] | 213,874 |
| 13 | Asthma/ | 144,729 |
| 14 | Wheez$.mp. [mp=title, book title, abstract, original title, name of substance word, subject heading word, floating sub-heading word, keyword heading word, organism supplementary concept word, protocol supplementary concept word, rare disease supplementary concept word, unique identifier, synonyms, population supplementary concept word, anatomy supplementary concept word] | 16,676 |
| 15 | bronchial hyperresponsiveness.mp. [mp=title, book title, abstract, original title, name of substance word, subject heading word, floating sub-heading word, keyword heading word, organism supplementary concept word, protocol supplementary concept word, rare disease supplementary concept word, unique identifier, synonyms, population supplementary concept word, anatomy supplementary concept word] | 3,040 |
| 16 | bronchial hyperreactivity.mp. [mp=title, book title, abstract, original title, name of substance word, subject heading word, floating sub-heading word, keyword heading word, organism supplementary concept word, protocol supplementary concept word, rare disease supplementary concept word, unique identifier, synonyms, population supplementary concept word, anatomy supplementary concept word] | 9,073 |
| 17 | Bronchial Hyperreactivity/ | 7,578 |
| 18 | Rhinitis/ | 15,669 |
| 19 | Rhinitis.mp. [mp=title, book title, abstract, original title, name of substance word, subject heading word, floating sub-heading word, keyword heading word, organism supplementary concept word, protocol supplementary concept word, rare disease supplementary concept word, unique identifier, synonyms, population supplementary concept word, anatomy supplementary concept word] | 54,086 |
| 20 | Hayfever.mp. [mp=title, book title, abstract, original title, name of substance word, subject heading word, floating sub-heading word, keyword heading word, organism supplementary concept word, protocol supplementary concept word, rare disease supplementary concept word, unique identifier, synonyms, population supplementary concept word, anatomy supplementary concept word] | 376 |
| 21 | (hay adj fever).mp. [mp=title, book title, abstract, original title, name of substance word, subject heading word, floating sub-heading word, keyword heading word, organism supplementary concept word, protocol supplementary concept word, rare disease supplementary concept word, unique identifier, synonyms, population supplementary concept word, anatomy supplementary concept word] | 4,059 |
| 22 | Rhinitis, Allergic, Perennial/ | 7,656 |
| 23 | Rhinitis, Allergic, Seasonal/ | 14,133 |
| 24 | Rhinoconjunctivitis.mp. [mp=title, book title, abstract, original title, name of substance word, subject heading word, floating sub-heading word, keyword heading word, organism supplementary concept word, protocol supplementary concept word, rare disease supplementary concept word, unique identifier, synonyms, population supplementary concept word, anatomy supplementary concept word] | 2,772 |
| 25 | Conjunctivitis/ | 6,698 |
| 26 | Conjunctivitis.mp. [mp=title, book title, abstract, original title, name of substance word, subject heading word, floating sub-heading word, keyword heading word, organism supplementary concept word, protocol supplementary concept word, rare disease supplementary concept word, unique identifier, synonyms, population supplementary concept word, anatomy supplementary concept word] | 20,350 |
| 27 | Forced Expiratory Volume/ | 28,935 |
| 28 | Vital Capacity/ | 17,585 |
| 29 | Spirometry/ | 10,234 |
| 30 | Lung function.mp. [mp=title, book title, abstract, original title, name of substance word, subject heading word, floating sub-heading word, keyword heading word, organism supplementary concept word, protocol supplementary concept word, rare disease supplementary concept word, unique identifier, synonyms, population supplementary concept word, anatomy supplementary concept word] | 15,987 |
| 31 | Peak Expiratory Flow Rate/ | 5,798 |
| 32 | Forced expiratory volume.mp. [mp=title, book title, abstract, original title, name of substance word, subject heading word, floating sub-heading word, keyword heading word, organism supplementary concept word, protocol supplementary concept word, rare disease supplementary concept word, unique identifier, synonyms, population supplementary concept word, anatomy supplementary concept word] | 42,671 |
| 33 | FEV1.mp. [mp=title, book title, abstract, original title, name of substance word, subject heading word, floating sub-heading word, keyword heading word, organism supplementary concept word, protocol supplementary concept word, rare disease supplementary concept word, unique identifier, synonyms, population supplementary concept word, anatomy supplementary concept word] | 31,625 |
| 34 | "FEV 1".mp. [mp=title, book title, abstract, original title, name of substance word, subject heading word, floating sub-heading word, keyword heading word, organism supplementary concept word, protocol supplementary concept word, rare disease supplementary concept word, unique identifier, synonyms, population supplementary concept word, anatomy supplementary concept word] | 6,493 |
| 35 | "FEV0.5".mp. [mp=title, book title, abstract, original title, name of substance word, subject heading word, floating sub-heading word, keyword heading word, organism supplementary concept word, protocol supplementary concept word, rare disease supplementary concept word, unique identifier, synonyms, population supplementary concept word, anatomy supplementary concept word] | 104 |
| 36 | "FEV 0.5".mp. [mp=title, book title, abstract, original title, name of substance word, subject heading word, floating sub-heading word, keyword heading word, organism supplementary concept word, protocol supplementary concept word, rare disease supplementary concept word, unique identifier, synonyms, population supplementary concept word, anatomy supplementary concept word] | 62 |
| 37 | Forced vital capacity.mp. [mp=title, book title, abstract, original title, name of substance word, subject heading word, floating sub-heading word, keyword heading word, organism supplementary concept word, protocol supplementary concept word, rare disease supplementary concept word, unique identifier, synonyms, population supplementary concept word, anatomy supplementary concept word] | 15,593 |
| 38 | FV.mp. [mp=title, book title, abstract, original title, name of substance word, subject heading word, floating sub-heading word, keyword heading word, organism supplementary concept word, protocol supplementary concept word, rare disease supplementary concept word, unique identifier, synonyms, population supplementary concept word, anatomy supplementary concept word] | 12,077 |
| 39 | Peak expiratory flow rate.mp. [mp=title, book title, abstract, original title, name of substance word, subject heading word, floating sub-heading word, keyword heading word, organism supplementary concept word, protocol supplementary concept word, rare disease supplementary concept word, unique identifier, synonyms, population supplementary concept word, anatomy supplementary concept word] | 7,336 |
| 40 | PEF.mp. [mp=title, book title, abstract, original title, name of substance word, subject heading word, floating sub-heading word, keyword heading word, organism supplementary concept word, protocol supplementary concept word, rare disease supplementary concept word, unique identifier, synonyms, population supplementary concept word, anatomy supplementary concept word] | 6,525 |
| 41 | poll?nosi$.mp. [mp=title, book title, abstract, original title, name of substance word, subject heading word, floating sub-heading word, keyword heading word, organism supplementary concept word, protocol supplementary concept word, rare disease supplementary concept word, unique identifier, synonyms, population supplementary concept word, anatomy supplementary concept word] | 2,131 |
| 42 | SAR.mp. [mp=title, book title, abstract, original title, name of substance word, subject heading word, floating sub-heading word, keyword heading word, organism supplementary concept word, protocol supplementary concept word, rare disease supplementary concept word, unique identifier, synonyms, population supplementary concept word, anatomy supplementary concept word] | 25,221 |
| 43 | (pollen adj3 allergy).mp. [mp=title, book title, abstract, original title, name of substance word, subject heading word, floating sub-heading word, keyword heading word, organism supplementary concept word, protocol supplementary concept word, rare disease supplementary concept word, unique identifier, synonyms, population supplementary concept word, anatomy supplementary concept word] | 2,236 |
| 44 | Immunoglobulin.mp. [mp=title, book title, abstract, original title, name of substance word, subject heading word, floating sub-heading word, keyword heading word, organism supplementary concept word, protocol supplementary concept word, rare disease supplementary concept word, unique identifier, synonyms, population supplementary concept word, anatomy supplementary concept word] | 370,928 |
| 45 | Total IgE.mp. [mp=title, book title, abstract, original title, name of substance word, subject heading word, floating sub-heading word, keyword heading word, organism supplementary concept word, protocol supplementary concept word, rare disease supplementary concept word, unique identifier, synonyms, population supplementary concept word, anatomy supplementary concept word] | 5,620 |
| 46 | Immunoglobulin E/ | 46,299 |
| 47 | Dermatitis, Atopic/ | 26,854 |
| 48 | Neurodermatitis.mp. [mp=title, book title, abstract, original title, name of substance word, subject heading word, floating sub-heading word, keyword heading word, organism supplementary concept word, protocol supplementary concept word, rare disease supplementary concept word, unique identifier, synonyms, population supplementary concept word, anatomy supplementary concept word] | 1,846 |
| 49 | Neurodermatitis/ | 1,622 |
| 50 | atopic disease.mp. [mp=title, book title, abstract, original title, name of substance word, subject heading word, floating sub-heading word, keyword heading word, organism supplementary concept word, protocol supplementary concept word, rare disease supplementary concept word, unique identifier, synonyms, population supplementary concept word, anatomy supplementary concept word] | 2,282 |
| 51 | atopic dermatitis.mp. [mp=title, book title, abstract, original title, name of substance word, subject heading word, floating sub-heading word, keyword heading word, organism supplementary concept word, protocol supplementary concept word, rare disease supplementary concept word, unique identifier, synonyms, population supplementary concept word, anatomy supplementary concept word] | 31,354 |
| 52 | eczema.mp. [mp=title, book title, abstract, original title, name of substance word, subject heading word, floating sub-heading word, keyword heading word, organism supplementary concept word, protocol supplementary concept word, rare disease supplementary concept word, unique identifier, synonyms, population supplementary concept word, anatomy supplementary concept word] | 26,535 |
| 53 | Eczema/ | 12,879 |
| 54 | skin prick test$.mp. [mp=title, book title, abstract, original title, name of substance word, subject heading word, floating sub-heading word, keyword heading word, organism supplementary concept word, protocol supplementary concept word, rare disease supplementary concept word, unique identifier, synonyms, population supplementary concept word, anatomy supplementary concept word] | 9,791 |
| 55 | atopy.mp. [mp=title, book title, abstract, original title, name of substance word, subject heading word, floating sub-heading word, keyword heading word, organism supplementary concept word, protocol supplementary concept word, rare disease supplementary concept word, unique identifier, synonyms, population supplementary concept word, anatomy supplementary concept word] | 12,236 |
| 56 | besniers prurigo.mp. [mp=title, book title, abstract, original title, name of substance word, subject heading word, floating sub-heading word, keyword heading word, organism supplementary concept word, protocol supplementary concept word, rare disease supplementary concept word, unique identifier, synonyms, population supplementary concept word, anatomy supplementary concept word] | 48 |
| 57 | anaphyla$.mp. [mp=title, book title, abstract, original title, name of substance word, subject heading word, floating sub-heading word, keyword heading word, organism supplementary concept word, protocol supplementary concept word, rare disease supplementary concept word, unique identifier, synonyms, population supplementary concept word, anatomy supplementary concept word] | 43,223 |
| 58 | dermatolog$ disease.mp. [mp=title, book title, abstract, original title, name of substance word, subject heading word, floating sub-heading word, keyword heading word, organism supplementary concept word, protocol supplementary concept word, rare disease supplementary concept word, unique identifier, synonyms, population supplementary concept word, anatomy supplementary concept word] | 1,058 |
| 59 | Ocular allerg$.mp. [mp=title, book title, abstract, original title, name of substance word, subject heading word, floating sub-heading word, keyword heading word, organism supplementary concept word, protocol supplementary concept word, rare disease supplementary concept word, unique identifier, synonyms, population supplementary concept word, anatomy supplementary concept word] | 786 |
| 60 | 1 or 2 or 3 or 4 or 5 or 6 or 7 or 8 or 9 or 10 or 11 or 12 or 13 or 14 or 15 or 16 or 17 or 18 or 19 or 20 or 21 or 22 or 23 or 24 or 25 or 26 or 27 or 28 or 29 or 30 or 31 or 32 or 33 or 34 or 35 or 36 or 37 or 38 or 39 or 40 or 41 or 42 or 43 or 44 or 45 or 46 or 47 or 48 or 49 or 50 or 51 or 52 or 53 or 54 or 55 or 56 or 57 or 58 or 59 | 1,008,181 |
| 61 | aborigin$.mp. [mp=title, book title, abstract, original title, name of substance word, subject heading word, floating sub-heading word, keyword heading word, organism supplementary concept word, protocol supplementary concept word, rare disease supplementary concept word, unique identifier, synonyms, population supplementary concept word, anatomy supplementary concept word] | 12,934 |
| 62 | indigenous.mp. [mp=title, book title, abstract, original title, name of substance word, subject heading word, floating sub-heading word, keyword heading word, organism supplementary concept word, protocol supplementary concept word, rare disease supplementary concept word, unique identifier, synonyms, population supplementary concept word, anatomy supplementary concept word] | 2,184 |
| 63 | torres strait island$.mp. [mp=title, book title, abstract, original title, name of substance word, subject heading word, floating sub-heading word, keyword heading word, organism supplementary concept word, protocol supplementary concept word, rare disease supplementary concept word, unique identifier, synonyms, population supplementary concept word, anatomy supplementary concept word] | 4,881 |
| 64 | australian indigen$.mp. [mp=title, book title, abstract, original title, name of substance word, subject heading word, floating sub-heading word, keyword heading word, organism supplementary concept word, protocol supplementary concept word, rare disease supplementary concept word, unique identifier, synonyms, population supplementary concept word, anatomy supplementary concept word] | 560 |
| 65 | Australoid$.mp. [mp=title, book title, abstract, original title, name of substance word, subject heading word, floating sub-heading word, keyword heading word, organism supplementary concept word, protocol supplementary concept word, rare disease supplementary concept word, unique identifier, synonyms, population supplementary concept word, anatomy supplementary concept word] | 26 |
| 66 | first nation$.mp. [mp=title, book title, abstract, original title, name of substance word, subject heading word, floating sub-heading word, keyword heading word, organism supplementary concept word, protocol supplementary concept word, rare disease supplementary concept word, unique identifier, synonyms, population supplementary concept word, anatomy supplementary concept word] | 7,144 |
| 67 | first people.mp. [mp=title, book title, abstract, original title, name of substance word, subject heading word, floating sub-heading word, keyword heading word, organism supplementary concept word, protocol supplementary concept word, rare disease supplementary concept word, unique identifier, synonyms, population supplementary concept word, anatomy supplementary concept word] | 134 |
| 68 | 61 or 62 or 63 or 64 or 65 or 66 or 67 | 20,852 |
| 69 | 60 and 68 | 445 |

**Supplemental Figure 1.** The pattern of the number of publications on allergic diseases and asthma over time among Aboriginal and Torres Strait Islander people.

**Supplemental Table 3.** Study settings of the included studies.

| **Settings** | **Number** |
| --- | --- |
| Metropolitan, rural, and remote | 1 |
| Remote, homeless/long grass respondents | 1 |
| Central desert and from the tropical north | 1 |
| Rural, remote, city, and regional communities | 1 |
| Metropolitan/City | 1 |
| Rural | 1 |
| Remote, town camp, and urban area | 1 |
| Metropolitan, regional (inner/outer), rural, remote, and very remote | 1 |
| Regional | 1 |
| Urban and regional | 1 |
| Non-remote rural towns and rural areas | 2 |
| National | 3 |
| Major cities, Inner regional, Outer regional, Remote, very remote | 2 |
| Regional and rural | 2 |
| Urban | 2 |
| Rural and urban | 2 |
| Rural and remote region | 3 |
| Regional and remote area | 5 |
| Remote and very remote | 7 |
| Not reported/specified or not clear | 17 |

**Supplemental Table 4.** Characteristics of included studies that reported eczema.

| **Author and year** | **Design and setting** | **Participant’s characteristics** | **Outcome and asthma/allergic disease measurement** | **Main Findings** | **Comparison with non-Indigenous Australians** |
| --- | --- | --- | --- | --- | --- |
| Glasgow et al. 2003 | **Design:** Cross-sectional  **Study period:** 1999, 2000 and 2001  **Setting:** Population-based  **Urban/rural status:** Metropolitan/City  **State:** ACT  **Sources of funding**: The Australian Capital Territory (ACT) Department of Health and Community Care. | **Population:** Aboriginal and Torres Strait Islander people and non-Indigenous Australians **Inclusion criteria:** (i) All new entrant primary schoolchildren aged 4–6 years in 1999, 2000 and 2001 and (ii) Year 1–6 primary schoolchildren **Exclusion criteria:**  Not reported  **Total sample:** 10821  **Indigenous Australians:** 217  **Overall response rate:** 80% for Part 1, 72% for Part 2 for kindergarten children, 52% for the Year 1–6 survey, and 98% for Part 2 return rate for children with asthma or respiratory symptoms  **Mean age:** Not reported  **Other characteristics**: Not reported | **Primary outcome**: Respiratory symptom  **Was asthma/allergic disease an outcome:** No  **Available asthma/allergic disease-related data**: eczema  **Asthma/allergic disease-related measurement:** Self-reported | Prevalence of eczema was 25% ($p$= 0.03 for the comparison between Aboriginal and Torres Strait Islander people and non-Indigenous Australians) | Prevalence of eczema was 32% |
| Weber et al. 2019 | **Design:** Cross-sectional  **Study period:** January and November 2014  **Setting:** Community-based (specifically school-based)  **Urban/rural status:** regional and rural  **State:** TAS  **Source of funding:** The Clifford Craig Research  Trust | **Population:** Aboriginal and Torres Strait Islander people and non-Indigenous Australians **Inclusion criteria:** Students in grades 1 and 2 (average ages 6–8 years) **Exclusion criteria:** Not reported  **Total sample:** 1075  **Indigenous Australians:** 114  **Overall response rate:** 55.8%  **Median age (IQR)=** 8 years (7.6-8.6)  **Other characteristics**: Not reported | **Primary outcome**: Asthma and allergic disorders  **Was asthma/allergic disease an outcome:** Yes  **Available asthma/allergic disease-related data**:  Ever eczema diagnosis  **Asthma/allergic disease-related measurement:** self-reported | Prevalence of ever eczema diagnosis was 44.4%  (p =0.05 for the comparison between Aboriginal and Torres Strait Islander people and non-Indigenous Australians). | Prevalence of ever eczema diagnosis was 35.0% |
| Haggett et al. 2021 | **Design:** Cross sectional  **Study period:** January 2012 and January 2017  **Setting:** Primary Country Health Service visiting dermatology specialist  **Urban/rural status:** Remote  **State:** WA  **Source of funding:** Not reported. | **Population:** Aboriginal and Torres Strait Islander people and non-Indigenous Australians  **Inclusion criteria:** All outpatients seen by the primary WA Country Health Service visiting dermatology specialist **Exclusion criteria:**  Not reported  **Total sample:** 1459  **Indigenous Australians:** 311  **Overall response rate**: N/A  **Mean age:**  Not reported  **Other characteristics**: Not reported | **Primary outcome**: Dermatological disease  **Was asthma/allergic disease an outcome:** Yes  **Asthma/allergic disease-related data**: Eczema  **Available asthma/allergic disease-related measurement:** Diagnoses were categorised according to the Specialised Content Topic Areas of the Australasian College of Dermatologists dermatology training programme curriculum | Prevalence of eczema/dermatitis was 19% | Prevalence of eczema/dermatitis was 17% |
| Hall et al. 2017 | **Design:** Cross-sectional  **Study period:** February 2013 and October 2015  **Setting:** Community Health Centre  **Urban/rural status:** Urban  **State:** QLD  **Source of funding:** Queensland Children’s Medical Research Institute project grant, a University of Queensland Foundation Research Excellence Award, a Queensland Government Smart Futures Fellowship (51008) and a QUT  Indigenous Health start-up research grant. | **Population:** Aboriginal and Torres Strait Islander people **Inclusion criteria:** Children aged less than 5 years **Exclusion criteria:**  Not reported  **Total sample:** 180  **Indigenous Australians:** 180  **Overall response rate**: 71.4%  **Median (IQR):** 18.4 months (7.7-35)  **Other characteristics**: 51% male and 49% female | **Primary outcome**: Acute respiratory illness  **Was asthma/allergic disease an outcome:** No  **Available asthma/allergic disease-related data**: History of eczema  **Asthma/allergic disease-related measurement:** Self-reported | History of life-time eczema was 13.4%. | N/A |
| Heyes et al. 2011 | **Design:** Cross sectional  **Study period:**  1 January 2010–31 July 2010  **Setting:** Hospital  **Urban/rural status:**  Not reported  **State:** WA  **Source of funding:** Not reported | **Population:** Aboriginal and Torres Strait Islander people and non-Indigenous Australians **Inclusion criteria:** All patients attended dermatology clinics over the study period. **Exclusion criteria:**  Not reported  **Total sample:**  4873  **Indigenous Australians:** 104  **Overall response rate:** N/A  **Median age (IQR):**  22 (7, 43)  **Other characteristics**: 40.4% male and 59.6% female | **Primary outcome**: Dermatological conditions  **Was asthma/allergic disease an outcome:** Yes  **Available asthma/allergic disease-related data**: Eczematous conditions  **Asthma/allergic disease-related measurement:**  Based on medical record | Prevalence of eczema was 12.5%. | N/A |
| Tilakaratne et al. 2016 | **Design:** Cross sectional  **Study period:** February to July 2013  **Setting:** Mixed: Hospital, Outreach and Outpatient encounter  **Urban/rural status:** Rural and urban  **State:** SA and NT  **Source of funding:** Not reported | **Population:** Aboriginal and Torres Strait Islander people and non-Indigenous Australians **Inclusion criteria:** Outpatient encounters were prospectively recorded from all patients seen by a single trainee during outpatient clinics in the months of February to July 2013 inclusive **Exclusion criteria:** Data from encounters where the trainee observed the dermatologist’s consultation with the patient were not included, even if the trainee was involved in the patient’s care. Patients’ inpatient, ward-consult and surgical encounters were not considered during this study period due to the lack of a comparable inpatient and surgical dermatology experience for the trainee at the outreach sites.  **Total sample:** 245 from urban, 213 from Outreach, and 458 total. In addition, 358 from Tertiary (Royal Adelid Hospital (RAH) and Royal Darwin Hospital (RDH)), 100 from Peripheral and 458 total.  **Indigenous Australians:** 2 (0.8%) from urban, 41 (19%) from Outreach, 43 (9%) total. And 8 (2%) from Tertiary (Royal Adelid Hospital and Royal Darwin Hospital), 35 (35%) from Peripheral and 43 (9%) total.  **Overall response rate**: N/A  **Mean age:** Not reported  **Other characteristics**: Not reported | **Primary outcome**: Dermatological disease  **Was asthma/allergic disease an outcome:** Yes  **Available asthma/allergic disease-related data**: Eczema/dermatitis  **Asthma/allergic disease-related measurement:** The 28 disease categories in the Australasian College of Dermatologists (ACD) curriculum and a 29^th^ category were recorded; instances where a skin check was carried out, but without a diagnosis of skin cancer or other incidental diagnosis being made, indicated in the results tables as a normal full skin examination (FSE). | Prevalence of eczema/dermatitis in urban and outreach areas was 7% in total with 0% from urban, and 7% from Outreach. The prevalence of eczema/dermatitis in Tertiary and Peripheral hospitals was 7% in total with 11% from Tertiary (RAH and RDH) and 6% from Peripheral. | N/A |
| Ricciardo et al. 2024 | **Design:** Cross-sectional cohort study  **Study period:** 4 to 8 October 2021  **Setting:** Aboriginal community-controlled health organisation  **Urban/rural status:** Metropolitan, regional (inner/outer), rural, remote, and very remote  **State:** WA  **Source of funding**: A Wesfarmers Centre of Vaccines and Infectious Diseases (WCVID) Seed Funding Grant. | **Population:** Aboriginal and Torres Strait Islander people **Inclusion criteria:** Aboriginal children (0–18 years) recruited from the waiting room. **Exclusion criteria:** Not reported  **Total sample:** 84  **Indigenous Australians:** 84  **Overall response rate:** 100%  **Median age (IQR):** 8 years (5,12)  **Other characteristics**: 44% male and 56% female | **Primary outcome**: Skin health and disease  **Was asthma/allergic disease an outcome:** Yes  **Available asthma/allergic disease-related data**: Eczema/dermatitis and  Other eczema/dermatitis (including irritant contact dermatitis, dyshidrotic eczema, juvenile plantar dermatosis,  lip-lickers dermatitis)  **Asthma/allergic disease-related measurement:** Based on past medical history and skin examination | Prevalence of eczema/dermatitis based on past medical history in urban living Aboriginal people was 19%, eczema/dermatitis based on skin examination was 15%, and other eczema/dermatitis (including irritant contact dermatitis, dyshidrotic eczema, juvenile plantar dermatosis,  lip-lickers dermatitis) based on skin examination was 8%. | N/A |
| Ronaldson et al. 2022 | **Design:** Cross sectional  **Study period:** 1 January 2020 to 31 December 2020.  **Setting:** Hospital  **Urban/rural status:** Regional  **State:** VIC  **Source of funding:** Not reported | **Population:** Aboriginal and Torres Strait Islander people and non-Indigenous Australians **Inclusion criteria:** All Emergency Department (ED) presentations between 1 January and 31 December 2020 via the Electronic Medical Records (EMR-Sunrise) database **Exclusion criteria:** Not reported  **Total sample:** 1484  **Indigenous Australians:** 39  **Overall response rate:** N/A  **Mean age:** Not reported  **Other characteristics**: Not reported | **Primary outcome**: Dermatology presentations to the ED  **Was asthma/allergic disease an outcome:** Yes  **Available asthma/allergic disease-related data**:  Allergy to skin (T784, T887, L509, T783) and  Eczema  **Asthma/allergic disease-related measurement:** Based on the International Classification of Diseases 10th revision (ICD-10) | Prevalence of eczema/dermatitis among >= 18 years was 7.6% and among <= 17 years was 14.8%.  Prevalence of allergy to skin among >=18 years participants was 5.1% and among <=17 years participants was 11.1%. | Prevalence of eczema/dermatitis among >= 18 years was 3.1% and among <= 17 years was 8.6%.  Prevalence of allergy among >=18 years participants was 11.1% and among <=17 years participants was 20.7%. |
| Blake et al. 2020 | **Design:** Cross-sectional  **Study period:** June 2015 and November 201  **Setting:** Community-based for the cross-sectional survey and hospitals and community primary health centres for the medical record  **Urban/rural status:** Rural and remote regions only  **State:** QLD and NT  **Sources of funding**: The Indigenous Respiratory Outreach Care Program received funding under the Queensland Aboriginal and Torres Strait Islander Health Investment Strategy for this work, through the Aboriginal and Torres Strait Islander Health Unit of Queensland Health. | **Population:** Aboriginal and Torres Strait Islander people **Inclusion criteria:** Aboriginal and Torres Strait Islander children and young adults (aged 3–25 years old). **Exclusion criteria:** Participants with incomplete data (missing ethnicity, self-reported responses and/or medical record information) and participants from city and inner regional locations as the study could not check all potentially relevant medical records from multiple primary care and specialist clinics in these locations.  **Total sample:** 889  **Indigenous Australians:** 889  **Overall response rate:** Not applicable (N/A)  **Mean age:** Not reported  **Other characteristics**: 49.7% male 50.3% female | **Primary outcome**: Asthma, Allergic rhinitis, and Eczema  **Was asthma/allergic disease an outcome:** Yes  **Available asthma/allergic disease-related data**: Asthma, Allergic rhinitis, and Eczema  **Asthma/allergic disease-related measurement:** Compare parent-/self-reported diagnoses with the one in the medical record. | Prevalence of eczema (ever) was 5.8% based on self-report data (SR), 6.2% based on medical records (MR), and 2.0% based on both SR and MR were considered. | Not applicable (N/A) |
| Collaro et al. 2021 | **Design:** Cross-sectional study  **Study period:** June 2015 and October 2017  **Setting:** Mixed (population-based and medical record review)  **Urban/rural status:** Regional/remote QLD and NT communities and schools.  **State:** QLD and NT  **Sources of funding:** Not reported. | **Population:** Aboriginal and Torres Strait Islander people **Inclusion criteria:** First Nations Australian children and young adults aged 5–25 years from the Indigenous Respiratory Reference Values (IRRV) stud**y Exclusion criteria:**  Not reported  **Total sample:** 909  **Indigenous Australians:** 909  **Overall response rate:** Of 1,276 participants, 355 (28%) were excluded due to unacceptable or missing spirometry, and 11 for unverifiable parent-reported pneumonia.  **Median age (IQR):** 11.0 (8.2, 13.8)  **Other characteristics**: 49.0% male and 51.0% female | **Primary outcome**: Lung function and asthma  **Was asthma/allergic disease an outcome:** No  **Available asthma/allergic disease-related data**: Eczema  **Asthma/allergic disease-related measurement:** Based on self-report | Overall prevalence of eczema was 5%, eczema with never-pneumonia was 5%, eczema with ever pneumonia was 6%, eczema with pneumonia occurred when < 3 years of age was 8%, eczema with pneumonia occurred when 3-5 years was 0, and eczema with first pneumonia occurred when > 5 year was 11% | N/A |

**Supplemental Table 5**. Characteristics of included studies that reported food reactions.

| **Author and year** | **Design and setting** | **Participant’s characteristics** | **Outcome and asthma/allergic disease measurement** | **Main findings** | **Comparison with non-Indigenous Australians** |
| --- | --- | --- | --- | --- | --- |
| Weber et al. 2019 | **Design:** Cross-sectional  **Study period:** January and November 2014  **Setting:** Community-based (specifically school-based)  **Urban/rural status:** regional and rural  **State:** TAS  **Source of funding:** The Clifford Craig Research  Trust | **Population:** Aboriginal and Torres Strait Islander people and non-Indigenous Australians **Inclusion criteria:** Students in grades 1 and 2 (average ages 6–8 years) **Exclusion criteria:** Not reported  **Total sample:** 1075  **Indigenous Australians:** 114  **Response rate**: 55.8%  **Median age (IQR)=** 8 years (7.6-8.6)  **Other characteristics**: Not reported | **Primary outcome**: Asthma and allergic disorders  **Was asthma/allergic disease an outcome:** Yes  **Asthma/allergic disease-related data**:  Food reaction ever  **Asthma/allergic disease-related measurement:** Self-reported | Prevalence of food reaction (ever) was 12.9% ($p$= 0.25 for the comparison between  Aboriginal and Torres Strait Islander people and non-Indigenous Australians). | Prevalence of food reaction (ever) was 16.8%. |

**Supplemental Table 6**. Characteristics of included studies that reported anaphylaxis.

| **Author and year** | **Design and setting** | **Participant’s characteristics** | **Outcome and asthma/allergic disease measurement** | **Main Findings** | **Comparison with non-Indigenous Australians** |
| --- | --- | --- | --- | --- | --- |
| Salter et al. 2020 | **Design:** Cross sectional  **Study period:** 2002-2013  **Setting:** Community/ population-based linked administrative data  **Urban/rural status:** Not reported  **State:** WA  **Source of funding:** The Western Australian Department of Health (G05767) and an unrestricted grant provided by Mylan Specialty Limited (IIT16-007). | **Population:** Aboriginal and Torres Strait Islander people and non-Indigenous Australians **Inclusion criteria:** Anaphylaxis events (emergency visit, admission, ambulance attendance or death) in the Western Australian anaphylaxis cohort (WAAC) subset, 2002–2013 **Exclusion criteria:** Transferred cases were excluded  **Total sample:** 12637  **Indigenous Australians:** Not clear  **Overall response rate**: N/A  **Mean age:** Not reported  **Other characteristics**: Not reported | **Primary outcome:** Anaphylaxis events, factors influencing occurrence, and change in event rates from 2002 to 2013  **Was asthma/allergic disease an outcome:** Yes  **Asthma/allergic disease-related data:** Anaphylaxis  **Asthma/allergic disease-related measurement:** Based on ICD-9-CM and ICD-10-AM | Overall anaphylaxis cases between 2002 and 2013 were 2.5%.  Trend of anaphylaxis: 3.3% in 2002, 2.5% in 2003, 2.2% in 2004, 2.1% in 2005, 2.3% in 2006, 1.4% in 2007, 3.5% in 2008, 1.5% in 2009, 2.5% in 2010, 3.1% in 2011, 2.7% in 2012, 2.6% in 2013. | Overall anaphylaxis cases between 2002 and 2013 were 97.5%.  Trend of anaphylaxis: 96.7% in 2002, 97.5% in 2003, 97.8% in 2004, 97.9% in 2005, 97.7% in 2006, 98.6% in 2007, 96.5% in 2008, 98.5% in 2009, 97.5% in 2010, 96.9% in 2011, 97.3% in 2012, 97.4% in 2013. |

**Supplemental Table 7**. Characteristics of included studies that reported allergic rhinitis.

| **Author and year** | **Design and setting** | **Participant’s characteristics** | **Outcome and asthma/allergic disease measurement** | **Main Findings** | **Comparison with non-Indigenous Australians** |
| --- | --- | --- | --- | --- | --- |
| Downs et al. 2001 | **Design:** Cross-sectional  **Study period:** July and August in Wagga Wagga and September in Moree, 1997  **Setting:** Population-based  **Urban/rural status:** Non-remote rural towns  **State:** NSW  **Sources of funding:** The National Health and Medical Research Council and Glaxo Allen+ Hanburys. | **Population:** Aboriginal and Torres Strait Islander people and non-Indigenous Australians  **Indigenous status:** Aboriginal people **Inclusion criteria:** Children aged seven to 12 years from school years three, four, five and six in Moree and three, four and five in Wagga Wagga whose parents gave written consent were included in the study**. Exclusion criteria:** Children who were of Torres Strait Islander origin were excluded from the analyses  **Total sample:** 1499  **Indigenous Australians:** 158  **Overall response rate:** 67.3%  **Mean age:** 9.4 + 1.1  **Other characteristics**: 48% male and 52% female | **Primary outcome**: Asthma and Hay fever  **Was asthma/allergic disease an outcome:** Yes  **Asthma/allergic disease-related data**: Hay fever  **Asthma/allergic disease-related measurement:** Parent-reported | Prevalence of hay fever in the last 12 months was 23.3% ($p<0.05)$.  Prevalence of ever having had hay fever or eczema was 31.4% ($p<0.05)$.  Risk factors for hay fever were parents' history of hay fever or eczema (Adjusted odds ratio (AOR)=17.9 (95% confidence interval (CI): 3.5-90.8)). | Prevalence of Hay fever in the last 12 months was 35.2%.  Prevalence of hay fever or eczema ever was 44.2%.  Risk factors for hay fever were atopy, parent history of hay fever or eczema, and bronchitis before age two *(effect size was not reported in the text but in the forest plot).* |
| Glasgow et al. 2003 | **Design:** Cross-sectional  **Study period:** 1999, 2000 and 2001  **Setting:** Population-based  **Urban/rural status:** Metropolitan/City  **State:** ACT  **Sources of funding:** The Australian Capital Territory (ACT) Department of Health and Community Care. | **Population:** Aboriginal and Torres Strait Islander people and non-Indigenous Australians **Inclusion criteria:** (i) all new entrant primary schoolchildren aged 4–6 years in 1999, 2000 and 2001, 217 being Indigenous children and 10 604 being non-indigenous children (80% of eligible); and (ii) Year 1–6 primary schoolchildren **Exclusion criteria:** Not reported  **Total sample:** 10821  **Indigenous Australians:** 217  **Response rate:** 80% for Part 1, 72% for Part 2 for kindergarten children, 52% for the Year 1–6 survey, and 98% for Part 2 return rate for children with asthma or respiratory symptoms  **Mean age:** Not reported  **Other characteristics**: Not reported | **Primary outcome**: Respiratory symptom  **Was asthma/allergic disease an outcome:** Yes  **Asthma/allergic disease-related data**: Hay fever  **Asthma/allergic disease-related measurement:** Self-reported | Prevalence of hay fever (ever) was 14.0%. | Prevalence of hay fever (ever) was 19.0%. |
| Weber et al. 2019 | **Design:** Cross-sectional  **Study period:** January and November 2014  **Setting:** Community-based (specifically school-based)  **Urban/rural status:** regional and rural  **State:** TAS  **Source of funding:** The Clifford Craig Research Trust | **Population:** Aboriginal and Torres Strait Islander people and non-Indigenous Australians  **Inclusion criteria:** Students in grades 1 and 2 (average ages 6–8 years) **Exclusion criteria:** Not reported  **Total sample:** 1075  **Indigenous Australians:** 114  **Response rate:** 55.8%  **Median age (IQR)=** 8 years (7.6-8.6)  **Other characteristics**: Not reported | **Primary outcome**: Asthma and allergic disorders  **Was asthma/allergic disease an outcome:** Yes  **Asthma/allergic disease-related data**: Allergic rhinitis ever and  hay fever ever  **Asthma/allergic disease-related measurement:** Self-reported | Prevalence of allergic rhinitis (ever) was 37.3%  Prevalence of hay fever (ever) was 22.6% | Prevalence of allergic rhinitis (ever) was 30.4%  Prevalence of hay fever (ever) was 24.2% |
| Ricciardo et al. 2024 | **Design:** Cross-sectional cohort study  **Study period:** 4 to 8 October 2021  **Setting:** Aboriginal community-controlled health organisation  **Urban/rural status:** Metropolitan, regional (inner/outer), rural, remote, and very remote  **State:** WA  **Source of funding**: A Wesfarmers Centre of Vaccines and Infectious Diseases (WCVID) Seed Funding Grant. | **Population:** Aboriginal and Torres Strait Islander people **Inclusion criteria:** Aboriginal children (0–18 years) recruited from the waiting room. **Exclusion criteria:** Not reported  **Total sample:** 84  **Indigenous Australians:** 84  **Response rate:** 100%  **Median age (IQR):** 8 (5,12)  **Other characteristics**: 44% male and 56% female | **Primary outcome**: Skin health and disease  **Was asthma/allergic disease an outcome: No**  **Asthma/allergic disease-related data**: Hay fever  **Asthma/allergic disease-related measurement:** Based on past medical history | Past medical history of hay fever in urban living Aboriginal people was 16.0%. | N/A |
| Blake et al. 2020 | **Design:** Cross-sectional (incorporating medical record review)  **Study period:** June 2015 and November 201  **Setting:** Community-based for the cross-sectional survey and hospitals and community primary health centres for the medical record  **Urban/rural status:** Rural and remote regions only  **State:** QLD and NT  **Sources of funding**: The Indigenous Respiratory Outreach Care Program received funding under the Queensland Aboriginal and Torres Strait Islander Health Investment Strategy for this work, through the Aboriginal and Torres Strait Islander Health Unit of Queensland Health. | **Population:** Aboriginal and Torres Strait Islander people **Inclusion criteria:** Aboriginal and Torres Strait Islander children and young adults (aged 3–25 years old). **Exclusion criteria:** Participants with incomplete data (missing ethnicity, self-reported responses and/or medical record information) and participants from city and inner regional locations as the study could not check all potentially relevant medical records from multiple primary care and specialist clinics in these locations.  **Total sample:** 889  **Indigenous Australians:** 889  **Overall response rate:** N/A  **Mean age:** Not reported  **Other characteristics**: 49.7% male and 50.3% female | **Primary outcome**: Asthma, Allergic rhinitis, and Eczema  **Was asthma/allergic disease an outcome:** Yes  **Asthma/allergic disease-related data**: Asthma, Allergic rhinitis, and Eczema  **Asthma/allergic disease-related measurement:** Compare parent-/self-reported diagnoses with the one in the medical record. | Prevalence of allergic rhinitis (ever) was 6.6% based on self-report (SR), 0.6% based on medical record (MR), and 0.2% based on both SR and MR. | N/A |
| Collaro et al. 2021 | **Design:** Cross-sectional study  **Study period:** June 2015 and October 2017  **Setting:** Mixed (population-based and medical record review)  **Urban/rural status:** Regional/remote QLD and NT communities and schools.  **State:** QLD and NT  **Sources of funding:** Not reported. | **Population:** Aboriginal and Torres Strait Islander people **Inclusion criteria**: Aboriginal and Torres Strait Islander children and young adults aged 5–25 years from the Indigenous Respiratory Reference Values (IRRV) stud**y Exclusion criteria:** Not reported  **Total sample:** 909  **Indigenous Australians:** 909  **Overall response rate:** Of 1,276 participants, 355 (28%) were excluded due to unacceptable or missing spirometry, and 11 for unverifiable parent-reported pneumonia.  **Median age (IQR):** 11.0 (8.2, 13.8)  **Other characteristics**: 49.0% male and 51.0% female | **Primary outcome**: Lung function and asthma  **Was asthma/allergic disease an outcome:** No  **Asthma/allergic disease-related data**: Hay fever  **Asthma/allergic disease-related measurement:** Based on self-report | Prevalence of hay fever with all subjects was 6.0%, with never-pneumonia was 6.0%, with ever-pneumonia was 12.0%, with pneumonia occurring when at age < 3 years was 15.0%, with pneumonia occurring between 3 to 5 years was 0.0%, and with first pneumonia occurred at age > 5 years was 0.0%. | N/A |

**Supplemental Table 8**. Characteristics of included studies that reported atopy.

| **Author and year** | **Design and setting** | **Participants characteristics** | **Outcome and asthma/allergic disease measurement** | **Main Findings** | **Comparison with non-Indigenous Australians** |
| --- | --- | --- | --- | --- | --- |
| Bremner et al. 1998 | **Design:** Cross-sectional  **Study period:** 1998 (No specified date and month specified)  Study participants  **Setting:** Population-based  **Urban/rural status:** Not reported  **State:** WA  **Sources of funding**: The Medical Research Fund of Western Australia (MEDWA). | **Population:** Aboriginal and Torres Strait Islander people **Inclusion criteria:** All available residents 5 years of age or older who were present during the 10-day period of the study were invited to participate. **Exclusion criteria:** Not reported  **Total sample:** 207  **Indigenous Australians:** 207  **Overall response rate:** 100%  **Mean age**: Not reported  **Other characteristics**: 46.4% male and 53.6% female | **Primary outcome**: Respiratory Symptoms and Lung Function  **Was asthma/allergic disease an outcome:** Yes  **Asthma/allergic disease-related data**: Atopy (any allergen, house dust mite, grass pollens, cat/dog dander, and moulds)  **Asthma/allergic disease-related measurement:** Atopy was measured by skin prick test (SPT) reactions to a panel of allergens (house dust mite, cat and dog dander, ryegrass, and moulds [Hol-lister-Stier, Spokane, WA]) applied to the forearm. | Prevalence of  atopy (SPT>2 mm):  (1) Under 18-year male: any allergen (18.9%), house dust mite (18.9%), grass pollens (0.0%), cat/dog dander (0.0%), moulds (0.0%)  (2) Under 18-year female: Any allergen (22.2%), House dust mite (17.8%), grass pollens (4.4%), cat/dog dander (2.2%), moulds (2.2%)  (3) >= 18-year male: Any allergen (28.8%), House dust mite (28.8%), grass pollens (3.4%), cat/dog dander (1.7%), moulds (1.7%)  (4) >=18 years female: Any allergen (36.4%), House dust mite (27.3%), grass pollens (6.1%), cat/dog dander (4.5%), moulds (4.5%) | Prevalence of atopy (SPT>2 mm):  (1) Under 18-year male: any allergen (54.9%), house dust mite (47.2%), grass pollens (33.5%), cat/dog dander (23.6%), moulds (20.6%)  (2) Under 18-year female: Any allergen (45.5%), house dust mite (33.6%), grass pollens (30.2%), cat/dog dander (16.2%), moulds (17.9%)  (3) >= 18-year male: Any allergen (53.1%), House dust mite (43.3%), grass pollens (33.2%), cat/dog dander (25.3%), moulds (19.1%)  (4) >=18 years female: Any allergen (52.4%), House dust mite (42.9%), grass pollens (36.0%), cat/dog dander (26.2%), moulds (16.0%) |
| Downs et al. 2001 | **Design:** Cross-sectional  **Study period:** July and August in Wagga Wagga and September in Moree, 1997  **Setting:** Population-based  **Urban/rural status:** Non-remote rural towns  **State:** NSW  **Sources of funding:** The National Health and Medical Research Council and Glaxo Allen+ Hanburys. | **Population:** Aboriginal and Torres Strait Islander people and non-Indigenous Australians **Inclusion criteria:** Children aged seven to 12 years from school years three, four, five and six in Moree and three, four and five in Wagga Wagga whose parents gave written consent were included in the study**. Exclusion criteria:** Children who were of Torres Strait Islander origin were excluded from the analyses  **Total sample:** 1499  **Indigenous Australians:** 158  **Overall response rate:** 67.3%  **Mean age:** 9.4 + 1.1  **Other characteristics**: 48% male and 52% female | **Primary outcome**: Asthma and Hay fever  **Was asthma/allergic disease an outcome:** No  **Asthma/allergic disease-related data**: Atopy  **Asthma/allergic disease-related measurement:** Sensitisation to eight allergens was measured by SPT on the forearm. | Prevalence of atopy was 36.2%, atopy to Alternaria was 7.2%, atopy to ray grass pollen was 9.2%, and atopy to house dust was 29.0%. | Prevalence of atopy was 45.6%, atopy to Alternaria was 20.5%, atopy to ray grass pollen was 27.3%, and atopy to house dust was 30.0%. |
| Veale et al. 1996. | **Design:** Cross-sectional  **Study period:** August 1990 and August/ September 1991.  **Setting:** Population-based study  **Urban/rural status:** Rural  **State:** QLD, SA, and NT  **Source of funding:** The Australian Institute of Aboriginal and  Torres Strait Islander Studies, the Queensland Asthma  Foundation and the Australian National University**.** | **Population:** Aboriginal and Torres Strait Islander people **Inclusion criteria:** All subjects aged five years and over in four rural Aboriginal communities were invited to answer a respiratory symptom questionnaire, have skin tests for common aeroallergens, and have a histamine bronchial challenge test. **Exclusion criteria:** Not reported  **Total sample:** 1252  **Indigenous Australians:** 1252  **Overall response rate:** The  participation rates in CY1, CY2, CA3  and CA4 were 80%, 51%, 72% and  87%, respectively.  **Mean age:** Not reported  **Other characteristics**: Not reported | **Primary outcome**: Atopy  **Was asthma/allergic disease an outcome:** Yes  **Asthma/allergic disease-related data**: Atopy  **Asthma/allergic disease-related measurement:** A wheal reaction of 3 mm or more to a SPT. | The overall prevalence of atopy was 30%, which was 33% in CY1 and 33% in CY2 where CY1 and CY2 are Aboriginal communities in Cape York (QLD), and 21% in CA3 and 34% in CA4, where CA3 and CA4 are Aboriginal communities in Central Australia.  Risk factors: Regional variation (P <0.00 1) and age (P< 0.001) (13% in 5-7 years, 21% in 8-12 years, 30% in 13-19 years, and 35% in 20-84 years). | N/A |

**Supplemental Table 9**. Characteristics of included studies that reported mixed allergies.

| **Author and year** | **Design and setting** | **Participant’s characteristics** | **Outcome and asthma/allergic disease measurement** | **Main findings** | **Comparison with non-Indigenous Australians** |
| --- | --- | --- | --- | --- | --- |
| Australian Bureau of Statistics, 2019. | **Design:** Survey  **Study period:** July 2018 until April 2019  **Setting:** Population-based  **Urban/rural status:**  **State:** National  **Source of funding:** The Australian Government Departments of Health and Prime Minister and Cabinet. | **Population:** Aboriginal and Torres Strait Islander people **Inclusion criteria:** Aboriginal and Torres Strait Islander peoples. **Exclusion criteria:** Non-Indigenous persons,  visitors to private dwellings staying for less than six months; people in households where all residents are less than 18 years of age; people who usually live in non-private dwellings, such as hotels, motels, hostels, hospitals, nursing homes and short-stay caravan parks; students at boarding school;  non-Australian diplomats, diplomatic staff and members of their household; members of non-Australian defence forces stationed in Australia and their dependents; and overseas visitors**.**  **Total sample:** 10,579 people from around 6,388 households.  **Indigenous Australians:** Same as above  **Overall response rate:** Of the 8,707 households in the final sample, 6,388 (73.4%) were fully or adequately responding households.  **Mean age:** Not reported  **Other characteristics**: Not reported | **Primary outcome**: Information about the health and wellbeing (health conditions, lifestyle  factors, health service use, social and emotional wellbeing, and physical measurements)  **Was asthma/allergic disease an outcome:** Yes  **Asthma/allergic disease-related data**: Foods, drugs, undefined allergy  **Asthma/allergic disease-related measurement:** Self-reported | Prevalence of foods, drugs, and undefined allergies:  Non-age standardised=10.3%  Age standardised=11.4%  Based on age category: 7.6% between 1-14 years old, 9.9% between 15-24 years old, 10.5% between 25-34 years, 10.8% between 35-44 years, 13.6% between 45-54 years, 14.8% 55+ years.  Based on sex:  Non-age standardised: 8.6% among males and 12.0% among females  Age-standardised: 8.7% among males and 13.7% among females. | Age-standardised prevalence of foods, drugs, and undefined allergies =12.2%  Based on sex:  Non-age standardised: 8.6% among males and 12.0% among females  Age-standardised: 9.5% among males and 14.7% among females. |
| Australian Bureau of Statistics, 2024. | **Design:** Survey  **Study period:** August 2022 to March 2024  **Setting:** Population-based  **Urban/rural status:**  **State:** National  **Source of funding:** The Australian Government Department of Health and Aged Care. | **Population:** Aboriginal and Torres Strait Islander people **Inclusion criteria:** Aboriginal and Torres Strait Islander peoples. **Exclusion criteria:** Non-Indigenous persons,  visitors to private dwellings staying for less than six months; people in households where all residents are less than 18 years of age; people who usually live in non-private dwellings, such as hotels, motels, hostels, hospitals, nursing homes and short-stay caravan park; students at boarding school;  non-Australian diplomats, diplomatic staff and members of their household; members of non-Australian defence forces stationed in Australia and their dependents; and overseas visitors**.**  **Total sample:** 7,768 people from around 4,878 households.  **Indigenous Australians:** Same as above  **Overall response rate:** Of the 7,839 households in the final sample, 4,878 (62.2%) were fully or adequately responding households.  **Mean age:** Not reported  **Other characteristics**: Not reported | **Primary outcome**: Information about the health and wellbeing (health conditions, lifestyle  factors, health service use, social and emotional wellbeing, and physical measurements)  **Was asthma/allergic disease an outcome:** Yes  **Asthma/allergic disease-related data**: Foods, drugs, undefined allergy  **Asthma/allergic disease-related measurement:** Self-reported | Prevalence of mixed allergies (foods, drugs, and undefined allergies) was 12.2%.  Based on age: 6.0% among 0-14, 14.2% among 15-24, 13.5% among 25-34, 13.4 among 35-44, 21.0 among 45-54, 15.4% among ≥55 years.  Based on sex: 14.7% among females and 9.5% among males.  Based on remoteness: 13.5% in non-remote areas and 4.2% in remote areas. | N/A |

**Supplemental Table 10**. Characteristics of included studies that reported asthma

| **Author and year** | **Design and setting** | **Participant’s characteristics** | **Outcome and asthma/allergic disease measurement** | **Main Findings** | **Comparison with non-Indigenous Australians** |
| --- | --- | --- | --- | --- | --- |
| Bremner et al. 1998 | **Design:** Cross-sectional  **Study period:** 1998 (No specified date and month specified)  **Setting:** population-based  **Urban/rural status:** Not reported  **State:** WA  **Sources of funding**: The Medical Research Fund of Western Australia (MEDWA). | **Population:** Aboriginal and Torres Strait Islander people  **Indigenous status:** Aboriginal people **Inclusion criteria:** All available residents 5 years of age or older who were present during the 10-day period of the study were invited to participate. **Exclusion criteria:** Not reported  **Total sample:** 207  **Indigenous Australians:** 207  **Overall response rate:** 100%  **Mean age:** Not reported  **Other characteristics**: 46.4% male and 53.6% female | **Primary outcome**: Respiratory Symptoms and Lung Function  **Was asthma/allergic disease an outcome:** Yes  **Asthma/allergic disease-related data**: Asthma  **Asthma/allergic disease-related measurement: S**elf-reported/a history of ever being diagnosed. | Prevalence of asthma (ever): (1) among < 18-year male was 5.4% and among < 18-year female was 8.9%; (2) among adult ≥ 18-year male was 8.5% and adult ≥ 18-year female was 15.2%. | Prevalence of asthma (ever): - (1) among < 18-year male was 29.2% and among < 18-year female was 19.6%; (2) among adult ≥ 18-year male was 15.5% and among adult ≥ 18-year female was 17.8%. |
| Brew et al. 2022 | **Design:** Prospective cohort with a data linkage: West Australia (WA) Birth Registrations, WA Death Registrations, WA Midwives Notification System (MNS), WA Hospital Morbidity Data Collection, and the WA Emergency Department Data Collection.  **Study period:** 2001-2013  **Setting:** Mixed settings  **Urban/rural status:** Urban, Inner region, Outer region, Remote, and Very remote  **State:** WA  **Sources of funding**: The National Health and Medical  Research Council of Australia (NHMRC project grant 1078214) and the NHMRC Centre for Research Excellence in Indigenous Adolescent and Child Health (GNT 1135273). | **Population:** Aboriginal and Torres Strait Islander people  **Inclusion criteria:** All singleton Aboriginal and Torres Strait Islander babies ≥20 weeks of gestation born in Western Australia (WA) from 2001 to 2013 to mothers who also identify as Indigenous**. Exclusion criteria:** Not reported  **Total sample:** 25484 Aboriginal and Torres Strait Islander children and 12323 Aboriginal and Torres Strait Islander mothers.  **Indigenous Australians:** 25484 Aboriginal and Torres Strait Islander children and 12323 Aboriginal and Torres Strait Islander mothers.  **Overall response rate**: N/A  **Mean age:** Not reported  **Other characteristics**: Not reported | **Primary outcome**: Perinatal outcomes  **Was asthma/allergic disease an outcome:** No  **Asthma/allergic disease-related data**: Maternal asthma  **Asthma/allergic disease-related measurement:** Maternal asthma was defined as answering yes to a question about having asthma during a prenatal visit (MNS) or being hospitalised or visiting an urban emergency department (ED) for asthma (principal diagnosis, International Statistical Classification of Diseases, Tenth Revision [ICD-10] code J45 or J46) during pregnancy or in the 3 years before pregnancy**.** | Prevalence of maternal asthma among all pregnancies was 11.0%, and, of these, 10.3% had an asthma exacerbation during pregnancy (1.3% of all pregnancies). | N/A |
| Cooksley et al. 2015 | **Design:** Cross-sectional  **Study period:** March 2008 to May 2012  **Setting:** Population-based  **Urban/rural status:** Remote and very remote area  **State:** WA  **Sources of funding:** The National Health and Medical Research Council (NHMRC) and the Thoracic Society of Australia and New Zealand (TSANZ) Robert Pierce Grant-in-Aid for Indigenous Lung Health. | **Population:**Aboriginal and Torres Strait Islander people and non-Indigenous Australians **Inclusion criteria:** Self-identified as either Aboriginal  and/or Torres Strait Islander or non-Indigenous Australians, and were permanently resident in the community, non-institutionalized and 40 years of age or older. **Exclusion criteria:** Not described  **Total sample:** 823  **Indigenous Australians:** 328  **Overall response rate:** 27.9% for Indigenous Australians and 20.8% for non-Indigenous Australians  **Mean age:** 51.1 + 8.8  **Other characteristics**: Not described | **Primary outcome**: Chronic obstructive pulmonary disease (COPD) and forced vital capacity (FVC)  **Was asthma/allergic disease an outcome:** No  **Asthma/allergic disease-related data**: Asthma or asthmatic bronchitis  **Asthma/allergic disease-related measurement:** self-report | Prevalence of asthma or asthmatic bronchitis with acceptable post-bronchodilator spirometry in Kimberley was 23.0% and with unacceptable post-bronchodilator spirometry in Kimberley was 16.2% | Prevalence of asthma or asthmatic bronchitis with acceptable post-bronchodilator spirometry in Kimberley and Australian non-Indigenous was 22.0% and 19.5%, respectively. prevalence of asthma or asthmatic bronchitis with unacceptable post-bronchodilator spirometry, in Kimberly and the Australian non-Indigenous was 27.1% and 14.0%, respectively. |
| Cunningham J. 2010 | **Design:** Cross-sectional (based on the National Aboriginal and Torres Strait Islander Health Survey)  **Study period:** 2004-05  **Setting:** Population-based  **Urban/rural status:** National survey  **State:** National  **Sources of funding:** The National Health and Medical Research Council Research Fellowship (#545200). | **Population:** Aboriginal and Torres Strait Islander people and non-Indigenous Australians **Inclusion criteria:** Aboriginal and Torres Strait Islander and non-Indigenous adults aged 18-64 years **Exclusion criteria:** Not presented  **Total sample:** 20849  **Indigenous Australians:** 5417  **Overall response rate:** Not reported as the study used secondary data.  **Mean age:** Not presented  **Other characteristics**: 46.8% male and 53.2% female | **Primary outcome**: Asthma  **Was asthma/allergic disease an outcome:** Yes  **Asthma/allergic disease-related data**: Asthma  **Asthma/allergic disease-related measurement:** Self-reported doctor or nurse diagnosed asthma that still exists. | Prevalence of asthma: 27.5% (95% CI 25.5-29.5) ever asthma and 16.2% (95% CI 14.6-17.8) current asthma  Risk factors: Non-English as a main language (AOR=0.5 (95% CI: 0.3-0.8)), Running out of food and couldn't afford to buy more in the last 12 months) (AOR=1.3, 95% CI: 1.0,1.7)), Remote or very remote area of residence (AOR=0.5, 95% CI: 0.3-.07)) | Prevalence of asthma: 20.6% (95% CI 19.7-21.5) ever asthma and 9.9% (95% CI 9.3-10.4) current asthma.  Risk factors: Non-English as a main language (AOR=0.3, 95% CI: 0.2-0.4), employment status not in the labour force (AOR=1.3, 95% CI:1.1,1.5)), Running out of food and couldn't afford to buy more in last 12 months (AOR=1.5, 95% CI=1.2, 2.0)), |
| Downs et al. 2001 | **Design:** Cross-sectional  **Study period:** July and August in Wagga Wagga and September in Moree, 1997  **Data source:** Survey  **Setting:** Population-based  **Urban/rural status:** Non-remote rural towns  **State:** NSW  **Sources of funding:** The National Health and Medical Research Council and Glaxo Allen+ Hanburys. | **Population:** Aboriginal and Torres Strait Islander people and non-Indigenous Australians  **Inclusion criteria:** Children aged seven to 12 years from school years three, four, five and six in Moree and three, four and five in Wagga Wagga whose parents gave written consent were included in the study**. Exclusion criteria:** Children who were of Torres Strait Islander origin were excluded from the analyses  **Total sample:** 1499  **Indigenous Australians:** 158  **Overall response rate:** 67.3%  **Mean age:** 9.4 + 1.1  **Other characteristics**: 48% male and 52% female | **Primary outcome**: Asthma and Hay fever  **Was asthma/allergic disease an outcome:** Yes  **Asthma/allergic disease-related data**: Asthma  **Asthma/allergic disease-related measurement:**  Asthma: Parent-reported (sneezing, running or blocked nose, sometimes with itchy eyes, not associated with cold)? If yes, was this in the last 12 months?). | Prevalence of asthma was 39.4%.  Risk factors for asthma: Parent has asthma (?), bronchitis before age two (AOR=19.3, 95% CI: 4.7, 79.3) | Prevalence of asthma WAS 39.3%.  Risk factors for asthma: atopy, parent has asthma, parent has hay fever or eczema, bronchitis before age two |
| Glasgow et al. 2003 | **Design:** Cross-sectional  **Study period:** 1999, 2000 and 2001  **Setting:** Population-based  **Urban/rural status:** Metropolitan/City  **State:** ACT  **Sources of funding:** The Australian Capital Territory (ACT) Department of Health and Community Care. | **Population:** Aboriginal and Torres Strait Islander people and non-Indigenous Australians **Inclusion criteria:** (i) all new entrant primary schoolchildren aged 4–6 years in 1999, 2000 and 2001, 217 being Indigenous children and 10 604 being non-indigenous children (80% of eligible); and (ii) Year 1–6 primary schoolchildren **Exclusion criteria:** Not presented  **Total sample:** 10821  **Indigenous Australians:** 217  **Response rate:** 80% for Part 1, 72% for Part 2 for kindergarten children, 52% for the Year 1–6 survey, and 98% for Part 2 return rate for children with asthma or respiratory symptoms  **Mean age:** Not reported  **Other characteristics**: Not reported | **Primary outcome**: Respiratory symptom  **Was asthma/allergic disease an outcome:** Yes  **Asthma/allergic disease-related data**: Asthma and hayfever  **Asthma/allergic disease-related measurement:** Self-reported | Prevalence of current asthma was 24%  Prevalence of asthma (ever) was 28%. | Prevalence of current asthma was 15%  Prevalence of asthma (ever) was 23%. |
| Hopkins et al. 2015 | **Design:** Cross-sectional survey  **Study period:** 2000-2002  **Setting:** Population-based  **Urban/rural status:** Urban  **State:** WA  **Source of funding:** Not reported | **Population:** Aboriginal and Torres Strait Islander people **Inclusion criteria:** Aboriginal youth (12–17 years) **Exclusion criteria:**  **Total sample:** 677 using weighted population estimates that allow for the complex survey design, this sample is weighted to an estimated population of 5,180 (95% CI: 5,130–5,180).  **Indigenous Australians:** 677  **Overall response rate**: Not reported.  **Mean age:** Not reported  **Other characteristics**: 50.4% were male and 49.6% were female | **Primary outcome**: Lifetime health problems and asthma symptoms  **Was asthma/allergic disease an outcome:** Yes  **Asthma/allergic disease-related data**: Asthma  **Asthma/allergic disease-related measurement:** self-reported | Prevalence of asthma symptoms was 50.5%.  Risk factors: Aboriginal youth with expected good (AOR=2.47, p <0.001) and resilient psychosocial status (AOR=1.76, p<.04) were significantly more likely to report no asthma symptoms than less Resilient youth. | N/A |
| Laird et al. 2022 | **Design:** Cross-sectional  **Study period:** March to November 2021  **Setting:** Population-based  **Urban/rural status:** Very remote  **State:** WA  **Source of funding**: Medical Research Future Fund and National and Mineral Resources Ltd | **Population:** Aboriginal and Torres Strait Islander people  **Inclusion criteria:** Aboriginal children aged <18 years in four different communities across two time points, 1‐month apart **Exclusion criteria:** Not reported  **Total sample:** 392  **Indigenous Australians:** 392  **Overall response:** 91.6%  **Median (IQR)=**8.4 years (5.1-11.5)  **Other characteristics**: 52.3% and 47.7% female | **Primary outcome**: Chronic respiratory disease  **Was asthma/allergic disease an outcome:** Yes  **Asthma/allergic disease-related data**: Asthma  **Asthma/allergic disease-related measurement:** Parent-reported current and past asthma | Prevalence of current/recent asthma was 4.3% and prevalence of previous asthma, that is no symptom or treatment in > 2 years was 0.8%.  Risk factors: Reports of asthma were relatively more frequent in communities serviced by a sealed road (p=0.02). | N/A |
| Markwick et al. 2014 | **Design:** Cross-sectional  **Study period:** 2008  **Setting:** Population-based  **Urban/rural status:** Not reported  **State:** VIC  **Source of funding:** Not reported | **Population:** Aboriginal and Torres Strait Islander people and non-Indigenous Australians **Inclusion criteria:** Adults, aged 18 years or older, who resided in private dwellings in Victoria and had access to a landline telephone. Only one person aged 18 years or older, per household, with the most recent birthday, was selected for interview. **Exclusion criteria**: Not reported  **Total sample:** 34168  **Indigenous Australians:** 339  **Overall response rate:** 65%  **Mean age:** Not reported  **Other characteristics**: Not reported | **Primary outcome**: Inequalities in the social determinants of health  **Was asthma/allergic disease an outcome:** No  **Asthma/allergic disease-related data**: Asthma  **Asthma/allergic disease-related measurement:** Self-reported doctor-diagnosed asthma | Prevalence of age-adjusted ever asthma was 29.3%, age adjusted current asthma was 16.4%. Prevalence ratio (95% CI) for having ever had asthma was 1.4 (1.1-1.8, p=0.018) and for current asthma was 1.6 (1.1-2.3, p=0.023). | Prevalence of age-adjusted ever asthma was 21.2%, age-adjusted current asthma was 10.7%. Prevalence ratio (95%CI) for non-Indigenous Australians study participants was assigned 1 (as a reference). |
| Melody et al. 2016 | **Design:** Cross-sectional (Environmental Health Needs Survey)  **Study period:** 2008  **Setting:** Community-based  **Urban/rural status:** Remote  **State:** WA  **Source of funding:** Not reported | **Population:** Aboriginal and Torres Strait Islander people  **Inclusion criteria:** Responses were obtained from either a community leader or community representative (typically a community elder or council member). Where appropriate, survey responses were completed on the basis of observation. Data were only analysed from communities that were identified as being ‘occupied’ at the time of the survey **Exclusion criteria:** Not reported  **Total sample:** 234  **Indigenous Australians:** 234  **Overall response rate:** Not clear  **Mean age:** Not reported  **Other characteristics**: Not reported | **Primary outcome**: Environmental health  **Was asthma/allergic disease an outcome:** No  **Asthma/allergic disease-related data**: Environmental factors associated with asthma/respiratory health issue  **Asthma/allergic disease-related measurement:** Based on self-reported responses from either a community leader or community representative (typically a community elder or council member). | Prevalence of asthma/respiratory health issue was 42.0%.  Risk factors: Dust was significantly associated with asthma/respiratory health issues (OR= 2.48 95 % CI: 1.43, 4.29). | N/A |
| Moore et al. 2007 | **Design:** Cross-sectional (Retrospective analysis of linked population data)  **Study period:** 1990-2000  **Setting:** Community/population-based  **Urban/rural status:** Metropolitan, rural, remote  **State:** WA  **Source of funding:** Not reported | **Population:** Aboriginal and Torres Strait Islander people and non-Indigenous Australians **Inclusion criteria:** All singleton live births in WA between 1 January 1990 and 31 December 2000 and their associated hospital admissions up to age 2 years were included. **Exclusion criteria:** Hospital birth records that were not associated with any illness and inter-hospital transfers were removed from the morbidity data set**.**  **Total sample:** 270071  **Indigenous Australians:** 17296  **Overall response rate:** N/A  **Mean age:** Not reported  **Other characteristics**: Not reported | **Primary outcome**: Hospital admission rates per 1000 live births for acute lower respiratory infection (ALRI) before age 2 years and linear time trends for ALRI admission rates  **Was asthma/allergic disease an outcome:** No  **Asthma/allergic disease-related data**: Temporal trends in admission for asthma  **Asthma/allergic disease-related measurement:** The Australian version of the 9th revision of the International Classification of Diseases (ICD9) was used for morbidity coding. Diagnoses since July 1999 have been coded using the 10th revision of the ICD and were converted into their best-match ICD9 code. | Temporal trends in admission for asthma: There was a 10%/year decline (47/1000 in 1992 to 18/1000 in 2000) before age 12 months and 7%/year (76 to 44/1000) for those aged 12–23 months (all P < 0.001). | Temporal trends in admission for asthma: There was a 7%/year (P < 0.001) decline before age 12 months (6/1000 live births in 1992 to 3/1000 in 2000) and 4%/year (P = 0.012) in children aged 12–23 months (20 to 13/1000). |
| Shepherd et al. 2012 | **Design:** Cross-sectional (Western Australian Aboriginal Child Health Survey)  **Study period:** 2000-2002  **Setting:** Community-based  **Urban/rural status:** Not reported  **State:** WA  **Source of funding:** Not reported | **Population:** Aboriginal and Torres Strait Islander people  **Inclusion criteria:** All Aboriginal children aged 0-17 years in in-scope families were selected to participate. **Exclusion criteria:** Not reported  **Total sample:** 5289  **Indigenous Australians:** 5289  **Overall response rate:** Not clear  **Mean age:** Not reported  **Other characteristics**: 51.6% (95%CI 49.9 53.1) males and 48.4% (95%CI 46.9 50.1) females | **Primary outcome**: Chronic conditions (asthma, sensory function problems, recurring chest infections and oral health problems) and acute conditions (ear infections and accidents and injuries).  **Was asthma/allergic disease an outcome:** Yes  **Asthma/allergic disease-related data**: Asthma  **Asthma/allergic disease-related measurement:** Primary carers reported, "ever had asthma" (Information on all health outcomes was gathered from primary carers of participating children). | Prevalence of asthma was 23.2% (95% CI 21.6, 24.9)  Risk factors: Index of Relative Indigenous Socioeconomic Outcomes (IRISEO) (Top quintile (AOR=9.2, 95% CI: 3.1, 27.2), Fourth (AOR=4.66, 95% CI: 2.80, 7.74), Third (AOR=3.91, 95% CI: 2.42, 6.31), and second (AOR=3.37, 95% CI: 2.07, 5.49) COMPARED to those in the bottom quintile; Neighbourhood characteristics (SEIFA quintiles) (Top quintile (AOR=3.48 95% CI: 1.34, 9.04), Third quintile (AOR=1.80, 95% CI: 1.29, 2.51), Second quintile (AOR=1.48, 95% CI: 1.10, 2.00) compared to those in bottom quintile; and Household tenure (Renting (AOR=1.90, 95% CI: 1.02, 3.53) compared to those who owned a house. | N/A |
| Trivedi et al. 2020 | **Design:** Quasi-experimental design  **Study period:** February 2006 to December 2009  **Urban/rural status:** Not reported  **Setting:** Population-based  **State:** NSW  **Source of funding:** Not reported | **Population:** Aboriginal and Torres Strait Islander people and non-Indigenous Australians  **Inclusion criteria:** Randomly selected participants from the general population who reported Aboriginal or Torres Strait Islander ethnicity **Exclusion criteria:** Not reported  **Total sample:** 267,153  **Indigenous Australians:** 1,948  **Overall response rate**: Not reported  **Unadjusted mean age**: 56.0 + 7.8 for registered and 58.3 + 9.3 for not registered**.**  **Propensity score weighted mean age:** 58.3 + 9.3 for registered and 58.4 + 9.3 for not registered  **Other characteristics**: Unadjusted females were 61.9% registered and 51.6% not registered. Propensity score weighted females were: 56.2% registered and 56.6% not registered | **Primary outcome**: A monthly measure of the number of prescriptions filled, monthly out-of-  pocket and government spending for medications and the monthly number of prescriptions filled for lipid-lowering, diabetes, and angiotensin-converting enzyme (ACE) inhibitor or angiotensin receptor blocker (ARB) medications  **Was asthma/allergic disease an outcome:** No  **Asthma/allergic disease-related data**: Asthma  **Asthma/allergic disease-related measurement:** Asthma status was collected from the study’s baseline survey (the 45 and Up study) and thus were not time-varying. | Unadjusted prevalence of asthma was 23.9% among registered for the copayment incentive and 10.8% among those not registered for the copayment incentive.  Propensity score weighted prevalence of asthma was 17.9% among those registered for the incentive and 17.7% among those not registered for the incentive. | N/A |
| Valery et al. 2008 | **Design:** Cross-sectional  **Study period:** 1999 and 2003  **Setting:** Population /community-based  **Urban/rural status:** Not reported  **State:** QLD  **Source of funding:** The Queensland Institute of Medical Research, Merck Sharpe and Dohme, a NHMRC project grant (ID 389838), a Rural Health Support, Education and Training (RHSET) grant, the Telstra Foundation and the Royal Children’s Hospital Foundation. | **Population:** Aboriginal and Torres Strait Islander people **Inclusion criteria:** All 5 to 17-year-olds enrolled at these schools were eligible to participate. Preferred respondents were the carers of 5–13-year-olds and the teenagers themselves for 13- to 17-year-olds. **Exclusion criteria:** Not described  **Total sample:** 682 in 1999 and 200 in 2003 on Thursday Island, 64 in 1999 and 43 in 2003 on Warraber Island. Overall: 200 on Thursday Island, 46 on Mabuiag Island, 26 on Horn Island, and 43 on Warraber Island.  **Indigenous Australians:** Similar to the total sample  **Overall response rate**: 361 children participated (34.4%)  and 315 (30%) answered the asthma questionnaire  (Thursday Island 24%, Mabuiag Island 94%, Horn  Island 23%, and Warraber Island 100%).  **Mean age:** Not reported  **Other characteristics**: Not reported | **Primary outcome**: Asthma  **Was asthma/allergic disease an outcome:** Yes  **Asthma/allergic disease-related data**: Asthma  **Asthma/allergic disease-related measurement:** Self-reported ever asthma | Overall prevalence of asthma was 12.2% with 14.2% in Thursday Island, 4.4% in Mabuiag Island, 30.8% in Horn Island, and 0% in Warraber Island.  Prevalence of asthma based on year and community was 16.9% in 1999 and 14.2% in 2003 in Thursday Island; 15.6% in 1999 and 0% in 2003 in Warraber Island. | N/A |
| Valery et al. 2003 | **Design:** Cross-sectional study  **Study period:** Not reported  **Setting:** Community/ population-based  **Urban/rural status:** rural and remote  **State:** QLD  **Source of funding:** Queensland Health, the Commonwealth Government, and the Sisters of Mercy–Mater Children’s Hospital. | **Population:** Aboriginal and Torres Strait Islander people and non-Indigenous Australians **Inclusion criteria:** All children between 3 and 17 years of age from five randomly selected Indigenous communities in the Torres Strait region**. Exclusion criteria:** Because of the difficulties in diagnosing asthma in under 3-year-olds, we have excluded them from this validation.  **Total sample:** 1327  **Indigenous Australians:** 1123  **Overall response rate:** 98%  **Mean age:** Not reported  **Other characteristics**: Not reported | **Primary outcome**: Asthma  **Was asthma/allergic disease an outcome:** Yes  **Asthma/allergic disease-related data**: Asthma  **Asthma/allergic disease-related measurement:** Children older than 6 years of age were clinically diagnosed as asthmatics if they had a history of recurrent wheeze and/or shortness of breath in the last 12 months, and were clinically responsive to a bronchodilator or had a 15% increase in forced expiratory volume in 1 sec (FEV1). Children aged 3 to 6 years were defined as having asthma if they had two or more episodes of wheezy illness that were associated with cough, shortness of breath, and documentation of improvement in symptoms or clinical signs after administration of bronchodilator were taken as evidence to support a diagnosis of asthma (even if not confirmed by spirometry). | Prevalence of asthma was 16.4% (95% CI: 14.2, 18.5). | Prevalence of asthma was 15.6% (95% CI: 9.1, 22.0). |
| Veale et al. 1996 | **Design:** Cross-sectional  **Study period:** August 1990 and August/ September 1991.  **Setting:** Population-based study  **Urban/rural status:** Rural  **State:** QLD, SA, and NT  **Source of funding:** The Australian Institute of Aboriginal and  Torres Strait Islander Studies, the Queensland Asthma  Foundation and the Australian National University**.** | **Population:** Aboriginal and Torres Strait Islander people **Inclusion criteria:** All subjects aged five years and over in four rural Aboriginal communities were invited to answer a respiratory symptom questionnaire, have skin tests for common aeroallergens, and have a histamine bronchial challenge test. **Exclusion criteria:** Not reported  **Total sample:** 1252  **Indigenous Australians:** 1252  **Overall response rate:** The participation rates in CY1, CY2, CA3 and CA4 were 80%, 51%, 72% and 87%, respectively.  **Mean age:** Not described  **Other characteristics**: Not described | **Primary outcome**:  Asthma  **Was asthma/allergic disease an outcome:** Yes  **Asthma/allergic disease-related data**: Asthma  **Asthma/allergic disease-related measurement:** The co-existence of recent wheeze and airway hyperresponsiveness (AHR); or recent wheeze and a greater than 15% increase in forced vital capacity (FEV) l after bronchodilator. | Overall current prevalence of asthma was 2.0% with 2.7% in CY1 and 0% in CY2, 1.0% in CA3 and 3.4% in CA4 (*where CY1 and CY2 are Aboriginal communities in Cape York (QLD), and CA3 and CA4are Aboriginal communities in Central Australia)*.  A factor associated with current asthma was an allergy to cats. The odds ratio for current asthma in subjects allergic to cats (controlling for house dust mite, alternaria and rye grass allergy) was 2.5 (95% CI, 1.3-5.0). There was significant regional variation in the prevalence of current asthma (P< 0.05). | N/A |
| Verheijden et al. 2002 | **Design:** Cross-sectional  **Study period:** Data from the tropical community were collected in May 1999 (dry season) and data from the central desert community were collected in September 2000 (dry season)  **Setting:** Population/ community based  **Urban/rural status:** Central desert and another  from the tropical north  **State:** WA  **Source of funding**: Not reported | **Population:** Aboriginal people **Inclusion criteria:** All AAD members of both communities who were 5 years of age or older and living in the community during the 10-day period of each study were invited to participate. **Exclusion criteria:** Not described  **Total sample:** 293  **Indigenous Australians:** 293  **Overall response rate:** Not reported  **Mean age:** Not reported  **Other characteristics**: Not reported | **Primary outcome**: Respiratory morbidity and lung function  **Was asthma/allergic disease an outcome:** Yes  **Asthma/allergic disease-related data**: Asthma  **Asthma/allergic disease-related measurement:** Self-reported physician/nurse diagnosed asthma (Asthma was determined from a positive response to the question: ‘Has a doctor/nurse ever told you that you have asthma?’) | The prevalence of asthma was 17% and 12% among < 18 years males and females, respectively, in Tropical. It was 7% and 20% among >=18 years males and females, respectively, in Tropical. It was 7% and 0% among <18 years male and females, respectively, in the Central desert, and 16% and 26% among ≥ 18 years males and females, respectively, in the Central desert. | N/A |
| Weber et al. 2019 | **Design:** Cross-sectional  **Study period:** January and November 2014  **Setting:** Community-based (specifically school-based)  **Urban/rural status:** regional and rural  **State:** TAS  **Source of funding:** The Clifford Craig Research Trust | **Population:** Aboriginal and Torres Strait Islander people and non-Indigenous Australians  **Inclusion criteria:** Students in grades 1 and 2 (average ages 6–8 years) **Exclusion criteria:** Not reported  **Total sample:** 1075  **Indigenous Australians:** 114  **Overall response rate**: 55.8%  **Median age (IQR)=** 8 years (7.6-8.6)  **Other characteristics**: Not reported | **Primary outcome**: Asthma and allergic disorders  **Was asthma/allergic disease an outcome:** Yes  **Asthma/allergic disease-related data**:  Asthma  **Asthma/allergic disease-related measurement:** Self-reported doctor diagnosis (ever). | The prevalence of doctor diagnosis asthma (ever) was 33.6% ($p value$= 0.04). | The prevalence of doctor diagnosis asthma (ever) was 24.9%. |
| MacIntyre et al 2005 | **Design:** Cross-sectional, randomised, computer-assisted telephone survey  **Study period:** 2000  **Setting:** Population-based  **Urban/rural status:** Not reported  **State:** NSW  **Source of funding:** Not reported | **Population:** Aboriginal and Torres Strait Islander people and non-Indigenous Australians  **Inclusion Criteria:** Household with at least one child 5-14 years old.  **Exclusion criteria:** Non-English-speaking carers  **Total sample:** 2020  **Indigenous Australians:** 34  **Overall response rate**: 41.9%  **Mean age:** Not reported  **Other characteristics:** Not reported | **Primary outcome:** Asthma and use of asthma medication  **Was asthma/allergic disease an outcome:** Yes  **Asthma/allergic disease-related data:** Asthma  **Asthma/allergic disease-related measurement:** Carer reported history of asthma diagnosis, hospital presentation/admission for asthma | The prevalence of asthma was 3.0% ($p value$ = 0.013). | The prevalence of asthma was 97.0%. |
| Australian Bureau of Statistics, 2019. | **Design:** Survey  **Study period:** July 2018 until April 2019  **Setting:** Population-based  **Urban/rural status:** National survey  **State:** National  **Source of funding:** The Australian Government Departments of Health and Prime Minister and Cabinet. | **Population:** Aboriginal and Torres Strait Islander people **Inclusion criteria:** Aboriginal and Torres Strait Islander peoples **Exclusion criteria:** Non-Indigenous persons  visitors to private dwellings staying for less than six months; people in households where all residents are less than 18 years of age; people who usually live in non-private dwellings, such as hotels, motels, hostels, hospitals, nursing homes and short-stay caravan park; students at boarding school; non-Australian diplomats, diplomatic staff and members of their household**;** members of non-Australian defence forces stationed in Australia and their dependents; and overseas visitors.  **Total sample:** 10,500 people from around 6,500 households.  **Indigenous Australians:** Same as above  **Overall response rate:** In non-community areas, 77% of households responded to screening, with 3.9% identifying an Aboriginal or Torres Strait Islander resident; 73% of these participated in the survey. In discrete Indigenous communities, 74.6% of in-scope households responded.  **Mean age:** Not reported  **Other characteristics**: Not reported | **Primary outcome**: Information about the health and wellbeing (health conditions, lifestyle  factors, health service use, social and emotional wellbeing, and physical  measurements)  **Was asthma/allergic disease an outcome:** Yes  **Asthma/allergic disease-related data**: Asthma  **Asthma/allergic disease-related measurement:** Self-reported | The overall lifetime prevalence of asthma was 15.7%, with 17.3% in non-remote areas, 8.6% in remote areas.  NSW: 19%  VIC: 18%  QLD: 14%  SA: 23%  WA: 13%  TAS: 22%  NT: 6%  ACT: 26% | N/A |
| Australian Bureau of Statistics, 2024. | **Design:** Survey  **Study period:** August 2022 to March 2024  **Setting:** Population-based  **Urban/rural status:** National survey  **State:** National  **Source of funding:** The Australian Government Department of Health and Aged Care. | **Population:** Aboriginal and Torres Strait Islander people **Inclusion criteria:** Aboriginal and Torres Strait Islander peoples. **Exclusion criteria:** Non-Indigenous persons  visitors to private dwellings staying for less than six months; people in households where all residents are less than 18 years of age; people who usually live in non-private dwellings, such as hotels, motels, hostels, hospitals, nursing homes and short-stay caravan park; students at boarding school; non-Australian diplomats, diplomatic staff and members of their household**;** members of non-Australian defence forces stationed in Australia and their dependents; and overseas visitors.  **Total sample:** 7,768 people from around 4,878 households.  **Indigenous Australians:** Same as above  **Overall response rate:** Of the 7,839 households in the final sample, 4,878 (62.2%) were fully or adequately responding households.  **Mean age:** Not reported  **Other characteristics**: Not reported | **Primary outcome**: Information about the health and wellbeing (health conditions, lifestyle  factors, health service use, social and emotional wellbeing, and physical  measurements)  **Was asthma/allergic disease an outcome:** Yes  **Asthma/allergic disease-related data**: Asthma  **Asthma/allergic disease-related measurement:** Self-reported | The overall lifetime prevalence of asthma was 16.6% with 18.2% in non-remote areas and 8.1% in remote areas. | N/A |
| Valery et al. 2001 | **Design:** Cross-sectional  **Study period:** August – October  1999  **Setting:** Population-based  **Urban/rural status:** Remote  **State:** QLD  **Source of funding:** A National  Health and Medical Research Council (NHMRC) project grant (ID 389838); a Rural Health Support, Education and Training grant; the Telstra Foundation,  and the Royal Children’s Hospital Foundation. | **Population:** Aboriginal and Torres Strait Islander children  **Inclusion criteria:** All 0 – 17-year-old children in the Torres Strait and Northern Peninsula Area of Australia  **Exclusion criteria:** Not described.  **Total sample:** 1,650  **Indigenous Australia:** 1,650  **Overall response rate**: 98.1%  **Mean age:** Not reported  **Other characteristics:** 53.4% Male and 46.6% Female | **Primary outcome:** Prevalence of asthma symptoms.  **Was asthma/allergic disease an outcome:** Yes  **Asthma/allergic disease-related measurement:** Self-reported | The overall prevalence of asthma was 15.8%, with 16.9% in Thursday  Island, 7.0% in Saibai  Island, 13.7% in Warraber  Island, 19.5% in Bamaga, and 7.7% in Umagico. | N/A |
| Al Alawi et al. 2021 | **Design:** Retrospective cohort study  **Study period:** 1 August 2008 and 31 December 2014  **Setting:** Hospital  **Urban/rural status:** Predominantly made up of rural and very remote communities  **State:** NT  **Sources of funding**: None | **Population:** Aboriginal and Torres Strait Islander people and non-Indigenous Australians  **Inclusion criteria:** All patients if hypomagnesaemia was listed as one of the diagnoses and a low serum level of magnesium could be confirmed. **Exclusion criteria:** Not reported.  **Total sample:** 876 **Indigenous Australians:** 494  **Overall response rate:** N/A  **Other characteristics**: 43.9% male | **Primary outcome**: Hypomagnesaemia  **Was asthma/allergic disease an outcome:** No  **Asthma/allergic disease-related data**: Bronchial Asthma  **Asthma/allergic disease-related measurement:** Not reported | Comorbidities of bronchial asthma among hospitalised patients was 12.4% ($p value=0.10$) | Comorbidities of bronchial asthma among hospitalised patients was 8.9% |
| Bisballe-Mülle et al. 2021 | **Design:** Comparative prospective cohort study  **Study period:** August 2017 and October 2019  **Setting:** Hospital  **Urban/rural status:** Not reported  **State:** NT  **Sources of funding**: None | **Population:** Aboriginal and Torres Strait Islander people and non-Indigenous Australians **Inclusion criteria:** Children aged from 2 weeks to <=16 years, unwell for 7 or fewer days, and hospitalised with one of the following illnesses; asthma, bronchiolitis, pneumonia, or other ARIs. Other ARIs were considered the diagnosis in children whose discharge diagnosis was nonspecific LRTI or URTI. Control subjects were eligible if they were aged 2 weeks to ≤16 years, hospitalised with a non-respiratory related illness, and were enrolled within the first 24 hours of admission to the paediatric ward. **Exclusion criteria:** If the potential study participants were previously enrolled  **Total sample:** 148  **Indigenous Australians:** 74  **Overall response rate:** N/A  **Mean age:** Not reported  **Other characteristics**: Not reported | **Primary outcome**: Acute cough (asthma, bronchiolitis, pneumonia, other acute respiratory infections)  **Was asthma/allergic disease an outcome:** Yes  **Asthma/allergic disease-related data:** Asthma  **Asthma/allergic disease-related measurement:** Asthma was diagnosed based on relatively standard clinical criteria i.e. clinical improvement of respiratory symptoms and/or signs (wheeze, tachypnea, dyspnea) to inhaled short-acting beta2 agonist. | The prevalence of asthma was 25% | The prevalence of asthma was 75% |
| Brew et al 2021 | **Design:** 12 Administrative and health data linkage: WA Birth Registrations, WA Death Registrations, WA Midwives Notification System (MNS), WA Hospital Morbidity Data Collection (HMDC), WA Emergency Department Data Collection (EDDC), WA Register of Developmental Anomalies (WARDA) and WA Mental Health Information System.  **Study period:** Between 2003 and 2012  **Setting:** Hospital  **Urban/rural status:** Major cities, Inner regional, Outer regional, Remote, very remote  **State:** WA  **Sources of funding: The** National Health and Medical Research Council of Australia (NHMRC Project grant 1078214) and the NHMRC Centre for Research Excellence in Aboriginal Adolescent and Child Health (GNT 1135273). | **Population:** Aboriginal and Torres Strait Islander people  **Inclusion criteria:** All Aboriginal children aged 1–4 years old born in WA between 2003 and 2012 **Exclusion criteria:** Children whose full siblings were not identified as Aboriginal.  **Total sample:** 25773  **Indigenous Australians:** 25773  **Overall response rate:** N/A  **Mean age:** Not reported  **Other characteristics**: 50.6% male and 49.4% female | **Primary outcome:** Asthma  **Was asthma/allergic disease an outcome:** Yes  **Asthma/allergic disease-related data:** Asthma  **Asthma/allergic disease-related measurement:** Asthma was identified from hospitalisation codes. | The prevalence of children hospitalised  for asthma at least once was 2.7%  Risk factors were being hospitalised for an acute respiratory tract infection (ARTI) (adjusted odds ratio (AOR)=4.06, 95% CI: 3.44, 4.78), area-level disadvantage (AOR= 1.58, 95% CI: 1.28, 1.94), being born at <33 weeks’ gestation (AOR=3.30, 95% CI: 2.52, 4.32) or birth weight <1500 g (AOR= 2.35, 95% CI: 1.39, 3.99). | N/A |
| Clifton et al. 2022 | **Design:** Retrospective Cohort (medical record review)  **Study period:** January 2008 to December 2019.  **Data source:** Routinely collected perinatal data  **Setting:** Hospital  **Urban/rural status:** Not described  **State:** QLD  **Sources of funding:** The NHMRC (AP1116640) and the UQ Research Training scholarship. | **Population:** Aboriginal and Torres Strait Islander people and non-Indigenous Australians **Inclusion criteria:** Asthmatic pregnant women with a singleton pregnancy **Exclusion criteria:** Not reported  **Total sample:** 18802  **Indigenous Australians:** 1118  **Overall response rate:** N/A  **Mean age:** Not reported  **Other characteristics**: Female: <20 years (11%), 20-34 years (75.7%), >=35 years (13.3%) | **Primary outcome**: Neonatal death  **Was asthma/allergic disease an outcome:** No  **Asthma/allergic disease-related data**: Asthma during pregnancy  **Asthma/allergic disease-related measurement:** Based on the medical record review | Prevalence of asthma was 23.9%  Risk factors: There was a statistically significant difference (p < 0.001) between Aboriginal and Torres Strait Islander women with asthma and non-Indigenous women with asthma based on age, education, smoking lifestyle, parity, mode of delivery, and maternal co-morbidity. | Prevalence of asthma was 16.7% |
| Collaro et al. 2021 | **Design:** Retrospective cohort study  **Study period:** February 2012-March 2020  **Data source:** Medical record review  **Setting:** Specialist clinic  **Urban/rural status:** Regional and Remote QLD  **State:** QLD  **Sources of funding:** Not reported. | **Population:** Aboriginal and Torres Strait Islander people and non-Indigenous Australians **Inclusion criteria:** Adults aged >18 years at first Indigenous Respiratory Outreach Care (IROC) clinic visit, who were medically reviewed and had spirometry and/or gas diffusion tests performed by a respiratory scientist. **Exclusion criteria:** Not reported  **Total sample:** 1761  **Indigenous Australians:** 1113  **Overall response rate**: N/A  **Median (IQR):** 55 (45-64)  **Other characteristics**: 38.0% male and 62.0% female | **Primary outcome**: Lung function  **Was asthma/allergic disease an outcome:** No  **Asthma/allergic disease-related data**: Asthma  **Asthma/allergic disease-related measurement:** based on medical record | Prevalence of asthma was 34.0% | Prevalence of asthma was 30.0% |
| Giarola et al. 2014 | **Design:** Cross sectional  **Study period:** January 2005 to December 2006  **Setting:** Hospital  **Urban/rural status:** Not described  **State:** NT  **Sources of funding:** A NHMRC Centre for Research Excellence in Lung Health of Aboriginal and Torres Strait Islander  Children (grant number 1040830). | **Population:** Aboriginal and Torres Strait Islander people and non-Indigenous Australians **Inclusion criteria:** Children ≤15 years of age admitted to Royal Darwin Hospital with asthma, diagnosed by the admitting paediatric team (wheezing illness responsive to salbutamol as  documented in the medical notes) were eligible. **Exclusion criteria:** Children observed in the emergency department and discharged home without admission to the hospital were excluded.  **Total sample:** 200  **Indigenous Australians:** 62  **Overall response rate:** N/A  **Median (IQR)=**4.3 (2.1-6.3)  **Other characteristics**: 37 out of 62 were male and 25 out of 62 were female. | **Primary outcome**: Factors associated with admission to acute asthma  **Was asthma/allergic disease an outcome:** Yes  **Asthma/allergic disease-related data**: Factors associated with admission for acute asthma  **Asthma/allergic disease-related measurement: B**ased on the International Classification of Diseases-10 codes J45-J46 for asthma diagnosis and self-report for the factors. | Factors associated with admission to acute asthma: 29% retrieved from the remote community (p <0.001). | Factors associated with admission to acute asthma: 5.8% retrieved from the remote community (p <0.001). |
| Heraganahally et al. 2023 | **Design:** Retrospective cohort study  **Study period:** Between 2010 and 2020  **Setting:** Hospital  **Urban/rural status:** Remote and regional Aboriginal communities referred to the respiratory and sleep outreach service by primary health practitioners and other health practitioners  **State:** NT  **Source of funding:** Division of  Medicine, Royal Darwin Hospital, Darwin, Australia. | **Population:** Aboriginal and Torres Strait Islander people **Inclusion criteria:** Patients identified to be currently  marked as ‘active patients’ as per the updated outreach records at the end of 2020, or marked as ‘active’ at the time of death if deceased prior to 2020. **Exclusion criteria:** Not reported  **Total sample:** 372  **Indigenous Australians:** 372  **Overall response rate**: N/A  **Median age (IQR):** 57.76 (50.95, 65.83)  **Other characteristics**: 36% male and 64% female | **Primary outcome**: Appropriateness of prescribing practice of inhaled pharmacotherapy  **Was asthma/allergic disease an outcome:** No  **Asthma/allergic disease-related data**: Asthma  **Asthma/allergic disease-related measurement:** Based on medical record review | Prevalence of asthma comorbidities was 16% | N/A |
| Heraganahally et al. 2022 | **Design:** Retrospective cohort study  **Study period:** Between 2012 and 2020  **Setting:** Hospital  **Urban/rural status:** Regional and remote  **State:** NT  **Source of funding:** Not reported. | **Population:** Aboriginal and Torres Strait Islander people and non-Indigenous Australians. **Inclusion criteria:** All patients aged 18 and above, who were identified to have had lung function testing (LFT) which were graded as acceptable and reproducible for session quality, and were assessed for BDR. **Exclusion criteria:** Patients who had multiple LFTs  **Total sample:** 5321  **Indigenous Australians:** 742  **Overall response rate:** N/A  **Median age (IQR):** 52.47 (45.03, 59.11) with BRD, 51.45 (42.42, 59.39) without BDR.  **Other characteristics**: Males were 48% for BDR and 42% without BDR. Females were 52% for BDR and 58% without BDR. | **Primary outcome**: Bronchodilator Responsiveness “Asthma”  **Was asthma/allergic disease an outcome:** Yes  **Asthma/allergic disease-related data**: Bronchodilator Responsiveness “Asthma”  **Asthma/allergic disease-related measurement:** The presence of “potential asthma” was considered if the study patients demonstrated BDR in the absence of evidence of either chronic obstructive pulmonary disease (COPD) or bronchiectasis on radiology. | Prevalence of bronchodilator responsiveness asthma was 17%  Risk factors: Indigenous patients with BDR were typically female, reported current smoking, and were a mean seven years younger, with a BMI a mean 2.6 units lower compared to non-Indigenous patients (P < 0.001). | Prevalence of bronchodilator responsiveness asthma was 13% |
| Heraganahall et al. 2019 | **Design:** Cross sectional  **Study period:** 2012–2016  **Setting:** Hospital  **Urban/rural status:** Regional and remote  **State:** NT  **Source of funding:** Not reported. | **Population:** Aboriginal and Torres Strait Islander people  **Inclusion criteria:** All adult Australian Aboriginal patients with a diagnosis of chronic obstructive pulmonary disease (COPD). Only patients from the regional and remote communities of the Top End NT of Australia referred to the specialist respiratory outreach team were included in this study. **Exclusion criteria:** Not described  **Total sample:** 380  **Indigenous Australians:** 380  **Overall response rate:** N/A  **Mean age:** 57.26 + 13.21  **Other characteristics**: 44.2% male and 55.8% female | **Primary outcome**: COPD  **Was asthma/allergic disease an outcome:** No  **Asthma/allergic disease-related data**: Asthma  **Asthma/allergic disease-related measurement:** Based on medical record review | Prevalence of asthma was 32.6% | N/A |
| Heraganahally et al. 2020 | **Design:** Cross-sectional  **Study period:** 2012-2016  **Setting:** Hospital  **Urban/rural status:** Regional and rural communities  **State:** NT  **Source of funding:** None | **Population:** Aboriginal and Torres Strait Islander people  **Inclusion criteria:** Only patients who had either a chest X-ray or computed tomography (CT) scan available to evaluate the presence or absence of bronchiectasis as per the reporting radiologist were included in the study. **Exclusion criteria:** Patients were excluded from the study if the clinical parameters were not consistent or documented in the clinical records for patients to have a diagnosis of COPD. Patients whose chest radiology (X-ray or CT scan) was not available to confirm the diagnosis of bronchiectasis were excluded from the analysis.  **Total sample:** 380  **Overall response rate:** N/A  **Indigenous Australians:** 380  **Mean age:** 58.4 + 12.1) (N=258)  **Other characteristics**: 50.4% male and 49.6% female | **Primary outcome**: COPD  **Was asthma/allergic disease an outcome:** No  **Asthma/allergic disease-related data**: Asthma  **Asthma/allergic disease-related measurement:** Based on medical record review. | Prevalence of overall asthma was 32.2%, asthma without bronchiectasis was 31.6%, and asthma with bronchiectasis was 33.3% | N/A |
| Howarth et al. 2024 | **Design:** Retrospective cohort (medical record review) study  **Study period:** 1 Jan 2011 – 31 Dec 2020  **Setting:** Hospital  **Urban/rural status:** Not reported  **State:** NT  **Source of funding:** The Thoracic Society of Australia and New Zealand (TSANZ) Robert Pierce Grant-In-Aid for Indigenous Lung Health. | **Population:** Aboriginal and Torres Strait Islander people  **Inclusion criteria:** Patients aged≥18 years diagnosed with bronchiectasis via chest Computed tomography (CT) scan between 2011 and 2020. **Exclusion criteria:** Not reported  **Total sample:** 459  **Indigenous Australians:** 459  **Overall response rate:** N/A  **Mean age:** Not reported  **Other characteristics**: Not reported | **Primary outcome**: Bronchiectasis  **Was asthma/allergic disease an outcome:** No  **Asthma/allergic disease-related data**: Asthma  **Asthma/allergic disease-related measurement:** Based on medical record review | Prevalence of asthma among bronchiectasis-admitted patients was 26.8% and asthma among not-admitted bronchiectasis patients was 18.0%. | N/A |
| Jayakody et al 2020 | **Design:** A historic cohort (medical record review) analysis  **Study period:** Between 2005/06 to 2013/14  **Setting:** Hospital  **Urban/rural status:** Not reported  **State:** NSW  **Source of funding:** The Priority Research Centre for Health Behaviour, University of Newcastle | **Population:** Aboriginal and Torres Strait Islander people and non-Indigenous Australians **Inclusion criteria:** Patients who: were aged 18 years and older at the time of index admission; were admitted to a NSW public hospital between 2005/6 and 2013/14; discharged from the hospital to the community (reflecting the focus on potentially avoidable admissions which are considered manageable through timely and effective community health care); and had one or more of the following selected ICD-10 defined ambulatory care sensitive (ACS) chronic conditions as a principle diagnosis: diabetic complications, asthma, angina, hypertension, congestive heart failure (CHF) and/or chronic obstructive pulmonary disease (COPD; including Bronchiectasis) **Exclusion criteria:** Private hospital admissions were excluded from the cohort. It was a priori acknowledged that most private hospital admissions are planned as very few private hospitals have emergency departments, and the majority of hospital admissions for Aboriginal people are in public hospitals (90%). Also, planned admissions were excluded.  **Total sample:** 27467  **Indigenous Australians:** 20306  **Overall response rate:** N/A  **Mean age:** 57.0 + 14.9  **Other characteristics**: 43.9% male and 56.1% female | **Primary outcome**: Number of avoidable admissions for an individual in each financial year  **Was asthma/allergic disease an outcome:** No  **Asthma/allergic disease-related data**: Asthma (as part of ambulatory care sensitive (ACS) chronic conditions as a principal diagnosis)  **Asthma/allergic disease-related measurement:** Based on medical record review | Prevalence of asthma as a principal diagnosis was 11.4%. | Prevalence of asthma as a principal diagnosis was 8.7%. |
| Mehra et al. 2021 | **Design:** Cross-sectional  **Study period:** 2012-2017  **Setting:** Hospital  **Urban/rural status:** Regional and remote  **State:** NT  **Source of funding:** Not reported**.** | **Population:** Aboriginal and Torres Strait Islander people and non-Indigenous Australians  **Inclusion criteria:** Adult patients diagnosed with bronchiectasis in the Top End Health Service (TEHS) region of the NT between 2012 to 2017 **Exclusion criteria:** Not reported  **Total sample:** 388  **Indigenous Australians:** 258  **Overall response rate:** N/A  **Median age (IQR)=** 54(44-64)  **Other characteristics**: 40.7% male and 50.3% female | **Primary outcome**: Bronchiectasis  **Was asthma/allergic disease an outcome:** No  **Asthma/allergic disease-related data**: Asthma  **Asthma/allergic disease-related measurement:** Based on medical record | Prevalence of asthma was 17.1% ($p value$= 0.875) | Prevalence of asthma was 17.7%. |
| Pal et al. 2022 | **Design:** Cross-sectional (face-to-face structured interview and retrospective review of electronic medical records)  **Study period:** July 2020 to October 2021  **Setting:** Mixed settings (hospital and respiratory outreach visits)  **Urban/rural status:** Urban (Darwin), Remote, homeless/long grass participants, but the study was conducted at Royal Darwin Hospital  **State:** NT  **Source of funding:** None | **Population:** Aboriginal and Torres Strait Islander people and non-Indigenous Australians **Inclusion criteria:** Patients were aged 18 years or older, residents of the TEHS region of the NT, with a clinically confirmed diagnosis of COPD. **Exclusion criteria:** Patients were not approached to participate or excluded if they were too unwell to carry out the interview, did not speak English at a level allowing communication, had mental health issues, that impeded their ability to participate in the interview, or did not give consent or withdrew consent during the interview process or did not want to continue with the interview.  **Total sample:** 86  **Overall response rate:** 86%  **Indigenous Australians:** 59  **Mean age:** 56.3 + 9.8  **Other characteristics**: 47% male and 53% female | **Primary outcome**: COPD disease knowledge, self-awareness and reasons for hospital presentations  **Was asthma/allergic disease an outcome:** No  **Asthma/allergic disease-related data**: Asthma  **Asthma/allergic disease-related measurement:** Based on medical records review. | Prevalence of asthma was 25% (p$value$ = 0.537). | Prevalence of asthma was 19%. |
| Ricciardo et al. 2024 | **Design:** Cross-sectional cohort study  **Study period:** 4 to 8 October 2021  **Setting:** Aboriginal community-controlled health organisation  **Urban/rural status:** Metropolitan, regional (inner/outer), rural, remote, and very remote  **State:** WA  **Source of funding**: A Wesfarmers Centre of Vaccines and Infectious Diseases (WCVID) Seed Funding Grant. | **Population:** Aboriginal and Torres Strait Islander people  **Inclusion criteria:** Aboriginal children (0–18 years) recruited from the waiting room. **Exclusion criteria:** Not reported  **Total sample:** 84  **Indigenous Australians:** 84  **Response rate:** 100%  **Median age (IQR):** 8 years (5,12)  **Other characteristics**: 44% male and 56% female | **Primary outcome**: Skin health and disease  **Was asthma/allergic disease an outcome:** No  **Asthma/allergic disease-related data**:  Asthma  **Asthma/allergic disease-related measurement:** Based on past medical history | Prevalence of asthma past medical history in urban living Aboriginal people was 11%. | N/A |
| Rothstein et al. 2007 | **Design:** Cross-sectional  **Study period:** June 2001 to February 2006  **Setting:** Paediatric Outreach Service  **Urban/rural status:** Remote  **State:** QLD  **Source of funding:** Not reported | **Population:** Aboriginal and Torres Strait Islander people and non-Indigenous **Inclusion criteria:** Children seen by the outreach service over the period June 2001 to February 2006 **Exclusion criteria:** Not reported  **Total sample:** 3562  **Indigenous Australians:** 819 (23%) Torres Strait Islander and 1994 (56%) Aboriginal peoples, [the remaining were non-Indigenous]  **Overall response rate:** N/A  **Mean age:** Not reported  **Other characteristics**: Not reported | **Primary outcome**: Number of children seen and common diagnoses  **Was asthma/allergic disease an outcome:** No  **Asthma/allergic disease-related data**: Asthma  **Asthma/allergic disease-related measurement:** Based on medical record review | The prevalence of asthma among Aboriginal and Torres Strait Islander children was 54 per 1000 and 40 per 1000, respectively, based on the total outreach practice population. Using the census population, the prevalence was 32 per 1000 and 10 per 1000, respectively. |  |
| Skinner et al. 2020 | **Design:** Cross-sectional analysis of a cohort study (population-based)  **Study period:** Between 2008 and 2011  **Setting:** Aboriginal community-controlled Health services  **Urban/rural status:** Urban and regional centres  **State:** NSW  **Source of funding**: A National Health and Medical  Research Council (NHMRC; grant IDs 358457, 512685,  1023998, 1035378, 1124822, 1135271), the NSW Ministry of  Health, the Australian Primary Health Care Research Institute,  beyondblue and the Rio Tinto Aboriginal Fund. | **Population:** Aboriginal and Torres Strait Islander people  **Inclusion criteria:** Aboriginal children attending these Aboriginal Community Controlled Health Services (ACCHSs) were eligible to participate in the study if their carer was aged 16 years or older and consented to follow-up interviews and linkage of baseline survey and clinical data to administrative health records. **Exclusion criteria:** Children aged less than 2 years, as carer-reported asthma in this age group may often correspond to transient wheezing associated with viral respiratory infections.  **Total sample:** 1247  **Indigenous Australians:** 1247  **Overall response rate:** Not clear  **Mean age:** Not reported  **Other characteristics**: 47.7% male and 52.3% female | **Primary outcome**: Asthma  **Was asthma/allergic disease an outcome:** Yes  **Asthma/allergic disease-related data**: Asthma  **Asthma/allergic disease-related measurement:** Children who had ever had asthma and children who had received treatment for asthma in the past month (recently treated asthma) were identified from carer responses to the question ‘Has your child ever had asthma?’ | Prevalence of individuals who have ever had asthma was 33.9% (95% credible interval (CrI)): 0.31 (0.28-0.34)) and recently treated asthma was 12.9% (95% CrI: 0.10 (0.08-0.13))  Prevalence estimates declined with increasing household income and increasing household size (posterior probabilities (PR) of decreasing trend >0.98), while children exposed to prenatal maternal smoking had a higher risk of asthma ever than unexposed children (PR 1.18 (95% credible interval 1.00–1.40)). Recently treated asthma prevalence was not significantly associated with pre-natal maternal (0.98 (0.71–1.41)) or current carer smoking (0.97 (0.68–1.37)); however, there was substantial uncertainty in PR estimates, and 95% credible intervals contained general-population estimates derived from the meta-analyses (1.37 (1.17–1.65) for prenatal smoking, 1.28 (1.15–1.44) for current parental or household smoking) | N/A |
| Thomas et al. 1998 | **Design:** Retrospective analysis (based on medical record review)  **Study period:** Samples were taken from two separate study weeks six months apart: one week in Darwin’s wet season (1-7 February 1994) and one in the dry season (9-15 August 1994)  **Setting:** Aboriginal community-controlled health service  **Urban/rural status:** Not reported  **State:** NT  **Source of funding**: Not reported | **Population:** Aboriginal and Torres Strait Islander people **Inclusion criteria:** All consultations from two separate study weeks six months apart: one week in Darwin’s wet season (1-7 February 1994) and one in the dry season (9-15 August 1994). Consultations with patients in nursing homes and aged care hostels and consultations after the first 200 for each study week were not excluded (unlike the Australian Morbidity and Treatment Survey (AMTS) (AMTS))  **Exclusion criteria:** Missing values were excluded from calculations of proportions.  **Total sample:** 583 in Danila Dilba and 98796 in AMTS  **Indigenous Australians:** 583  **Overall response rate:** N/A  **Mean age:** Not reported  **Other characteristics**: 44.1% male and 55.9% female | **Primary outcome**: Clinical consultation  **Was asthma/allergic disease an outcome:** No  **Asthma/allergic disease-related data**: Asthma  **Asthma/allergic disease-related measurement:** Based on the International Classification of Primary Care | Prevalence of asthma was 3.1% (95% CI: 1.7, 4.5) at Danila Dilba and 3.7% based on the Australian Morbidity and Treatment Survey. | N/A |
| Whybourne et al. 1999 | **Design**: Cross sectional  **Study period:** July 1991 to June 1997  **Setting:** Hospital  **Urban/rural status:** Urban and rural  **State:** NT  **Source of funding:** Not reported | **Population:** Aboriginal and Torres Strait Islander people and non-Indigenous Australians **Inclusion criteria:** All children aged 1–9 years residing in the Darwin urban or rural district. **Exclusion criteria:** Not described  **Total sample:** 477  **Indigenous Australians:** 100  **Overall response rate**: N/A  **Mean age:** Not reported  **Other characteristics**: Not reported | **Primary outcome**: Hospitalization for asthma  **Was asthma/allergic disease an outcome:** Yes  **Asthma/allergic disease-related data**: Asthma  **Asthma/allergic disease-related measurement:** Asthma was identified in children using the International Classification of Diseases Version 9 codes 493.0–493.9 inclusive**.** | Hospitalisation rate: As a principal diagnosis: 2.6/1000 population for Aboriginal and Torres Strait Islander people from rural areas, 4.7/1000 population for Aboriginal and Torres Strait Islander people children from urban areas (ATSI-urban) with 3.6/1000 overall rate. As comorbidity: 2.0/1000 population for Aboriginal and Torres Strait Islander people from rural areas, 1.3/1000 population for Aboriginal and Torres Strait Islander people children from urban areas with 1.7/1000 overall rate. Total (principal diagnosis and comorbidity): 4.6/1000 population for Aboriginal and Torres Strait Islander people from rural areas, 6.0/1000 population for Aboriginal and Torres Strait Islander people children from urban areas with 5.3/1000 overall rate.  The risk ratio for Indigenous Australian hospitalised children having asthma as a principal diagnosis compared to non-Indigenous Australians was 0.32 (0.26– 0.40) and 0.35 (0.29–0.42) as a comorbidity. The risk ratio for hospitalized rural and urban Indigenous Australian children having asthma as a principal diagnosis compared to non-Indigenous Australian was 0.16 (0.12-0.23) and 0.78 (0.60-1.03), respectively. The risk ratio for hospitalized rural and urban Indigenous Australian children having asthma as a principal diagnosis or comorbidity compared to non-Indigenous Australian was 0.21 (0.16-0.28) and 0.73 (0.58-0.93), respectively. The risk ratio for hospitalized rural children having asthma as a principal diagnosis compared to urban Indigenous Australian was 0.21 (0.14-0.32). The risk ratio for hospitalized rural children having asthma as a principal diagnosis or comorbidity compared to urban Aboriginal and Torres Strait Islander people was 0.29 (0.21-0.40). | As a principal diagnosis: 5.5/1000 population for non-Indigenous Australians (Note: no urban-rural data given).  As comorbidity: 1.9/1000 population for non-Indigenous Australians Total (principal diagnosis and comorbidity): 7.4/1000 population. |
| Williams et al. 1997 | **Design:** Retrospective analysis  **Study period:** 1988-1993  **Setting:** Hospital  **Urban/rural status:** Not reported  **State:** WA  **Source of funding:** Not reported | **Population:** Aboriginal and Torres Strait Islander people and non-Indigenous Australians **Inclusion criteria:** All hospital discharges for Aboriginals and non-Aboriginals in WA from 1988 through 1993 for which diseases of the respiratory system were the primary cause of hospitalization. **Exclusion criteria:** Not described  **Total sample:** Not clear  **Indigenous Australians:** Not clear  **Overall response rate**: N/A  **Mean age:** Not reported  **Other characteristics**: Not reported | **Primary outcome**: Respiratory tract diseases  **Was asthma/allergic disease an outcome:** Yes  **Asthma/allergic disease-related data**: Asthma  **Asthma/allergic disease-related measurement:** Based on the 9^th^ Edition of the International Classification of Diseases—Clinical  Modifications (ICD9-CM). | Asthma admission rate was 3348/100000 person-year among infants, 4175/100000 person-year among children aged 1-4 years old, 1092/100000 person-year among children aged 5-14 years old, 407/100000 person-year per individual aged 15-24 years old, 880/100000 person-year among individual aged 25-39 years old, 1835/100000 person-year among individual aged 40-54-year-olds, 2227/100000 person-year among individual aged 55+ years old. | Asthma admission rate was 635/100000 person-year among infants, 1975/100000 person-year among children aged 1-4 years old, 771/100000 person-year among children aged 5-14 years old, 250/100000 person-year per individual aged 15-24 years old, 132/100000 person-year among individual aged 25-39 years old, 159/100000 person-year among individual aged 40-54-year-olds, 358/100000 person-year among individual aged 55+ years old. |
| Einsiedel et al. 2014 | **Design:** Cross-sectional  **Study period:** Between 1 January 2000 and 31 December 2010.  **Setting:** Hospital  **Urban/rural status:** Remote, town camp, and urban area  **State/setting:** Central Australia region  **Source of funding**: The National Health and Medical Research Council (NHMRC project grant 1012945), the Northern Territory Rural Clinical School, which is an initiative of the Australian Department of Health and Ageing, and was also supported by grants from the Association pour la  Recherche sur le Cancer (ARC), the Cance´ropole/Ile de France, the CNRS (UMR 3569) and the Institut Pasteur, Paris, France. | **Population:** Aboriginal and Torres Strait Islander people  **Inclusion Criteria:** All adults (≥ 15 years) admitted to Alice Springs Hospital  **Exclusion criteria:** Patients whose western blot results were indeterminant  **Total sample:** 1451  **Indigenous Australians:** 1451  **Overall response rate**: N/A  **Mean age:** Not reported  **Other characteristics:** Not reported | **Primary outcome:** Human T-Lymphotropic Virus Type 1 infection (HTLV-1) and its clinical association  **Was asthma/allergic disease an outcome:** No  **Asthma/allergic disease-related data:** Asthma  **Asthma/allergic disease-related measurement:** Based on the International Classification of Diseases | HTLV-1 is associated with an increased risk of admission with asthma (coefficient, 0.99, 95% CI:0.27-1.701, p =0.007) | N/A |
| Blake et al. 2020 | **Design:** Cross-sectional (incorporating medical record review)  **Study period:** June 2015 and November 2011  **Setting:** Community-based for the cross-sectional survey and hospitals and community primary health centres for the medical record  **Urban/rural status:** Rural and remote regions only  **State:** QLD and NT  **Sources of funding**: The Indigenous Respiratory Outreach Care Program received funding under the Queensland Aboriginal and Torres Strait Islander Health Investment Strategy for this work, through the Aboriginal and Torres Strait Islander Health Unit of Queensland Health. | **Population:** Aboriginal and Torres Strait Islander people **Inclusion criteria:** Aboriginal and Torres Strait Islander children and young adults (aged 3–25 years old). **Exclusion criteria:** Participants with incomplete data (missing ethnicity, self-reported responses and/or medical record information) and participants from city and inner regional locations, as the study could not check all potentially relevant medical records from multiple primary care and specialist clinics in these locations.  **Total sample:** 889  **Indigenous Australians:** 889  **Overall response rate**: N/A  **Mean age:** Not reported  **Other characteristics**: 49.7% male and 50.3% female | **Primary outcome**: Asthma, Allergic rhinitis, and Eczema  **Was asthma/allergic disease an outcome: Yes**  **Asthma/allergic disease-related data**: Asthma, Allergic rhinitis, and Eczema  **Asthma/allergic disease-related measurement:** Compare parent-/self-reported diagnoses with the one in the medical record. | Prevalence of asthma was 15.7% based on self-report (SR), 10.3% based on medical record (MR), and 7.6% based on both SR and MR | N/A |
| Blake et al. 2020 | **Design:** Cross-sectional and medical record review  **Study period:** June 2015 and November 2017  **Setting:** Community-based (child care centres for 3-5-year-olds, schools and/or community groups for 5-17-year-olds and community events and groups and sporting groups) and health care centre and hospitals for the medical record  **Urban/rural status:** Rural and remote, city and regional communities  **State:** QLD and NT  **Sources of funding**: Funded by the Aboriginal and Torres Strait Islander Health Unit (Queensland Health) and supported by a National Health and Medical Research (NHMRC) Centre for Research Excellence  (CRE) grant for Indigenous Children (grant 1 040 830) and The Prince Charles Hospital Foundation New Investigator grant (NI2017-34). | **Population:** Aboriginal and Torres Strait Islander people **Inclusion criteria:** Male or female child/young adult who identified as Aboriginal, Torres Strait Islander or Aboriginal and Torres Strait Islander aged 3-25 years who had written and informed consent to participate; consent from parent/guardian if <18 years of age or by self if 18-25 years **Exclusion criteria:** Individuals with a known contraindication for performing spirometry and/or FeNO measurement: Heart attack, pneumothorax, pulmonary embolus or any surgery to chest, abdomen, brain, eyes, ears, nose or throat in the last 4-6 weeks and previously diagnosed with very high blood pressure (200/120mmHg) or an aortic aneurysm**.**  **Total sample:** 1245  **Indigenous Australians:** 1245  **Overall response rate**: Of 1287 recruited participants, medical history data was available for 1245 (97.4%) participants and reliable spirometry data for 1106 (86.5%) participants (n = 139 could not achieve spirometry results).  **Median age:** 10.5 (7.6-13.6)  **Other characteristics**: 49.5% male and 50.5% female | **Primary outcome**: Respiratory health profile  **Was asthma/allergic disease an outcome:** Yes  **Asthma/allergic disease-related data**: Asthma  **Asthma/allergic disease-related measurement:** Self-reported and medical record | Overall prevalence of asthma was 19.5%; with 23.5% in the City/Region, and 17.7% in Rural/Remote areas  Overall asthma-related hospitalisation was 3.1%, with 2.6% in the City/Region, and  3.4% in the Rural/Remote areas | N/A |
| Collaro et al. 2021 | **Design:** Cross-sectional study  **Study period:** June 2015 and October 2017  **Setting:** Mixed (population-based and medical record review)  **Urban/rural status:** Regional/remote QLD and NT communities and schools.  **State:** QLD and NT  **Sources of funding:** Not reported. | **Population:** Aboriginal and Torres Strait Islander people **Inclusion criteria:** First Nations Australian children and young adults aged 5–25 years from the Indigenous Respiratory Reference Values (IRRV) stud**y Exclusion criteria:** Not reported  **Total sample:** 909  **Indigenous Australians:** 909  **Response rate:** Of 1,276 participants, 355 (28%) were excluded due to unacceptable or missing spirometry, and 11 for unverifiable parent-reported pneumonia.  **Median age (IQR):** 11.0 (8.2, 13.8)  **Other characteristics**: 49.0% male and 51.0% female | **Primary outcome**: Lung function and asthma  **Was asthma/allergic disease an outcome:** Yes  **Asthma/allergic disease-related data**: Asthma/wheeze  **Asthma/allergic disease-related measurement:** Based on self-report and/or medical record | Prevalence of asthma/wheeze with all subjects was 13%; asthma with never-pneumonia was 12%; asthma with ever pneumonia was 23%; asthma with pneumonia occurred at age < 3 years was 23%; asthma with pneumonia occurred when 3-5 years was 8%; and asthma with first pneumonia occurred at age > 5 years 22%.  Risk factors: Ever-pneumonia status was associated with an increased risk of developing childhood asthma (AOR = 3.10, 95% CI: 1.49, 6.45).  When modelling was repeated with age of first pneumonia as the exposure, pneumonia first occurring before three (AOR = 2.67, 95% CI: 1.04, 6.85) and between three and five years (OR = 8.23, 95% CI: 2.73, 24.8) of age were associated with an increased risk of childhood asthma. | N/A |
| Hanigan et al. 2008 | **Design:** Data linkage  **Study period:** Between the 1st of April and the 30^th^ of November each year from 1996 to 2005  **Setting:** Population-based (linked with medical record)  **Urban/rural status:** Not reported  **State:** NT  **Source of funding:** Australian Research Council linkage grant  (grant number: LP0348543) with cash and in-kind support from the Northern Territory Government and Bureau of Meteorology. | **Population:** Aboriginal and Torres Strait Islander people and non-Indigenous Australians **Inclusion criteria:** Fire seasons between the 1st of April and the 30^th^ of November each year from 1996 to 2005 **Exclusion criteria:** Elective admissions and wet season  **Total sample:** 109,478  **Indigenous Australians:** 11,591  **Overall response rate:** N/A  **Mean age:** 11591  **Other characteristics**: Not reported | **Primary outcome**: Cardio-respiratory hospital admission  **Was asthma/allergic disease an outcome:** Yes  **Asthma/allergic disease-related data**: Asthma-related admission  **Asthma/allergic disease-related measurement:** Based on International Classification of Diseases (ICD) codes. | Asthma-related emergency hospitalisation was 18%. | Asthma-related emergency hospitalisation was 22%. |
| Homaira et al. 2017 | **Design:** Retrospective analysis using linked population-based administrative data.  **Study period:** 2000-2010  **Setting:** Mixed settings (linked data.)  **Urban/rural status:** Not reported  **State:** NSW  **Source of funding:** The Rotary Club of Sydney Cove and the  Cerebral Palsy Alliance of Australia. | **Population:** Aboriginal and Torres Strait Islander people and non-Indigenous Australians **Inclusion criteria:** 1. non-Indigenous high-risk children: Non-Indigenous children who (i) were born preterm (gestational age (GA) <37 weeks), (ii) were born at term with a birth weight of <2500 g.  2. Indigenous children: Children of mothers whose Indigenous status was recorded as Aboriginal and/or Torres Strait Islander in any of the data sets were considered to be Indigenous, including any born preterm or born with low birth weight.  3. Non-Indigenous standard risk children: All other non-Indigenous term children. **Exclusion criteria:** Not reported  **Total sample:** 847516  **Indigenous Australians:** 29692  **Overall response rate**: N/A  **Mean age:** 73 months ± 42 months  **Other characteristics**: 52% male and 48% female | **Primary outcome**: Risk of development of severe asthma in different subgroups of children who had RSV hospitalisation in the first 2 years of life compared with those who did not  **Was asthma/allergic disease an outcome:** Yes  **Asthma/allergic disease-related data**: Risk of development of severe asthma in different subgroups of children who had respiratory syncytial viral (RSV) hospitalisation in the first 2 years of life compared with those who did not  **Asthma/allergic disease-related measurement:** All hospitalisations with primary diagnostic codes associated with asthma (J45), predominantly  allergic asthma (J45.0), non-allergic asthma (J45.1), mixed asthma (J45.8), asthma unspecified (J45.9) and status asthmaticus (J46) or wheeze (R0.62) were considered as asthma hospitalisations. | Risk factors for first asthma hospitalisation: adjusted hazard ratio (AHR)=4.0 (95% CI: 3.3, 4.8) for 2–3 years of age. The adjusted HR  for first asthma hospitalisation was highest for children  between the ages 2 and 3 years  *(These findings compare the HR for first asthma hospitalisation beyond the age of 2 years in different subgroups of children (2-3, 3-5, 5-7, and >7) who had severe RSV*  *disease in the first 2 years of life compared with those who did not).* | Risk factors for first asthma hospitalisation:  AHR=3.9 (95% CI: 3.7, 4.1), for 2–3 years age for standard risk children and AHR= 4.3 (95% CI: 3.8, 4.9) for high-risk children. |

**Quality assessment**

**Supplemental Table 11**. JBI appraisal for studies reporting prevalence data

| Authors | Item 1 | Item 2 | Item 3 | Item 4 | Item 5 | Item 6 | Item 7 | Item 8 | Item 9 |
| --- | --- | --- | --- | --- | --- | --- | --- | --- | --- |
| Blake et al. 2020a | Yes | Yes | Yes | Yes | Yes | Yes | Yes | Yes | Yes |
| Blake et al. 2020b | Yes | Yes | Yes | Yes | Yes | Yes | Yes | Yes | Yes |
| Bremner et al. 1998 | Yes | Yes | Yes | Yes | Yes | Yes | No | Yes | Yes |
| Giarola et al. 2014 | Yes | Yes | No | Yes | Yes | Yes | Yes | Yes | N/A |
| Haggett et al. 2021 | Yes | Yes | Yes | Yes | Yes | Yes | Yes | Yes | N/A |
| Heraganahally et al. 2020 | Yes | Yes | Yes | Yes | Yes | Yes | Yes | Yes | N/A |
| Heyes et al. 2011 | Yes | Yes | No | Yes | Yes | Yes | Yes | Yes | N/A |
| Mehara et al. 2021 | Yes | Yes | Yes | Yes | Yes | Yes | Yes | Yes | N/A |
| Moore et al. 2007 | Yes | Yes | Yes | Yes | Yes | Yes | Yes | Yes | N/A |
| Pal et al. 2022 | Yes | No | No | Yes | Yes | Yes | Yes | Yes | Yes |
| Ricciardo et al. 2024 | Yes | No | Unclear | Yes | Yes | Yes | No | Yes | Yes |
| Ronaldson et al. 2022 | Yes | Yes | Yes | Yes | Yes | Yes | Yes | Yes | N/A |
| Rothstein et al. 2007 | Yes | Yes | Yes | Yes | Yes | Yes | Yes | Yes | N/A |
| Thomas et al. 1998 | Yes | Yes | Yes | Yes | Yes | Yes | Yes | Yes | N/A |
| Tilakaratne et al. 2016 | Yes | Yes | No | Yes | Yes | Yes | Yes | Yes | N/A |
| Verheijden et al. 2002 | Yes | Yes | No | Yes | Yes | Yes | Yes | Yes | Yes |
| Weber et al. 2019 | Yes | Yes | Yes | Yes | Yes | Yes | Yes | Yes | Yes |
| Whybourne et al. 1999 | Yes | Yes | Yes | Yes | Yes | Yes | Yes | Yes | N/A |
| Williams et al. 1997 | Yes | Yes | Yes | Yes | Yes | Yes | Yes | Yes | N/A |
| Australian Bureau of Statistics, 2019 | Yes | Yes | Yes | Yes | Yes | Yes | Yes | Yes | Yes |
| Australian Bureau of Statistics, 2024 | Yes | Yes | Yes | Yes | Yes | Yes | Yes | Yes | Yes |
| Valery et al. 2001. | Yes | Yes | Yes | Yes | Yes | Yes | Yes | Yes | Yes |

**Key (With answers: Yes, No, Unclear or Not Applicable)**

1. Was the sample frame appropriate to address the target population?

2. Were study participants sampled in an appropriate way?

3. Was the sample size adequate?

4. Were the study subjects and the setting described in detail?

5. Was the data analysis conducted with sufficient coverage of the identified sample?

6. Were valid methods used for the identification of the condition?

7. Was the condition measured in a standard, reliable way for all participants?

8. Was there appropriate statistical analysis?

9. Was the response rate adequate, and if not, was the low response rate managed appropriately?

**Supplemental Table 12**. JBI appraisal of analytical cross-sectional studies

| Authors | Item 1 | Item 2 | Item 3 | Item 4 | Item 5 | Item 6 | Item 7 | Item 8 |
| --- | --- | --- | --- | --- | --- | --- | --- | --- |
| Collaro et al. 2021 | Yes | Yes | Yes | Yes | Yes | Yes | Yes | Yes |
| Cooksley et al. 2015 | Yes | Yes | Yes | Yes | Yes | Yes | Yes | Yes |
| Cunningham et al 2010 | Yes | Yes | Yes | Yes | Yes | Yes | Yes | Yes |
| Downs et al. 2001 | Yes | Yes | Yes | Yes | Yes | Yes | Yes | Yes |
| Glasgow et al. 2003 | Yes | Yes | Yes | Yes | Yes | Yes | No | Yes |
| Hall et al. 2017 | Yes | Yes | Yes | Yes | Yes | Yes | Yes | Yes |
| Hanigan et al. 2008 | Yes | Yes | Yes | Yes | Yes | Yes | Yes | Yes |
| Heraganahally et al. 2022 | Yes | Yes | Yes | Yes | Yes | Yes | Yes | Yes |
| Heraganahally et al. 2019 | Yes | Yes | Yes | Yes | Yes | Yes | Yes | Yes |
| Hopkins et al 2015 | Yes | Yes | Yes | Yes | Yes | Yes | No | Yes |
| Laird et al. 2022 | Yes | Yes | Yes | No | Yes | Yes | Yes | Yes |
| Markwick et al. 2014 | Yes | Yes | Yes | Yes | Yes | Yes | Yes | Yes |
| Melody et al 2016 | Yes | Yes | Yes | No | Yes | Yes | No | Yes |
| Saltar et al. 2020 | Yes | Yes | Yes | Yes | Yes | Yes | Yes | Yes |
| Shepherd et al. 2012 | Yes | Yes | Yes | No | Yes | Yes | No | Yes |
| Skinner et al. 2020 | Yes | Yes | Yes | Yes | Yes | Yes | No | Yes |
| Valery et al. 2008 | Yes | Yes | No | Unclear | No | Yes | Unclear | Yes |
| Valery et al. 2003 | Yes | Yes | Yes | Yes | Yes | Yes | Yes | Yes |
| Veale et al. 1996 | Yes | Yes | Yes | Yes | Yes | Yes | Yes | Yes |
| Cagney et al. 2005 | Yes | Yes | Yes | Yes | Yes | Yes | No | Yes |
| Einsiedel et al. 2014 | Yes | Yes | Yes | Yes | Yes | Yes | Yes | Yes |

**Key (With answers: Yes, No, Unclear or Not Applicable)**

Item 1: Were the criteria for inclusion in the sample clearly defined?

Item 2: Were the study subjects and setting described in detail?

Item 3: Was the exposure measured in a valid and reliable way?

Item 4: Were objective, standard criteria used for measurement of the condition?

Item 5: Were confounding factors identified?

Item 6: Were strategies to deal with confounding factors stated?

Item 7: Were the outcomes measured in a valid and reliable way?

Item 8: Was appropriate statistical analysis used

**Supplemental Table 13**. JBI appraisal for cohort studies

| Authors | Item 1 | Item 2 | Item 3 | Item 4 | Item 5 | Item 6 | Item 7 | Item 8 | Item 9 | Item 10 | Item 11 |
| --- | --- | --- | --- | --- | --- | --- | --- | --- | --- | --- | --- |
| Al Alawi et al. 2022 | Yes | Yes | Yes | Yes | Yes | N/A | Yes | Yes | N/A | N/A | Yes |
| Bisballe-Mülle et al. 2021 | Yes | Yes | Yes | N/A | N/A | Yes | Yes | Yes | Yes | Unclear | Yes |
| Brew et al. 2021 | Yes | Yes | Yes | Yes | Yes | Yes | Yes | Yes | Yes | Unclear | Yes |
| Brew et al. 2022 | Yes | Yes | Yes | Yes | Yes | Yes | Yes | Yes | Yes | Unclear | Yes |
| Clifton et al. 2022 | Yes | Yes | Yes | Yes | Yes | Yes | Yes | Yes | Yes | N/A | Yes |
| Collaro et al. 2021 | Yes | Yes | Yes | Yes | Yes | Yes | Yes | Yes | Yes | N/A | Yes |
| Heraganahally et al. 2023 | Yes | Yes | Yes | Yes | Yes | N/A | Yes | Yes | N/A | N/A | Yes |
| Homaira et al. 2017 | Yes | Yes | Yes | Yes | Yes | Yes | Yes | Yes | Yes | Yes | Yes |
| Howarth et al. 2024 | Yes | Yes | Yes | Yes | Yes | Yes | Yes | Yes | No | N/A | Yes |
| Jayakody et al. 2020 | No | Yes | Yes | Yes | Yes | Yes | Yes | Yes | Yes | N/A | Yes |

**Key (With answers: Yes, No, Unclear or Not Applicable (N/A))**

Item 1: Were the two groups similar and recruited from the same population?

Item 2: Were the exposures measured similarly to assign people to both exposed and unexposed groups?

Item 3: Was the exposure measured in a valid and reliable way?

Item 4: Were confounding factors identified?

Item 5: Were strategies to deal with confounding factors stated?

Item 6: Were the groups/participants free of the outcome at the start of the study (or at the moment of exposure)?

Item 7: Were the outcomes measured in a valid and reliable way?

Item 8: Was the follow up time reported and sufficient to belong enough for outcomes to occur?

Item 9: Was follow up complete, and if not, were the reasons to loss to follow up described and explored?

Item 10: Were strategies to address incomplete follow up utilized?

Item 11: Was appropriate statistical analysis used?

**Supplemental Table 14.** JBI appraisal for quasi-experimental study

| Authors | Item 1 | Item 2 | Item 3 | Item 4 | Item 5 | Item 6 | Item 7 | Item 8 | Item 9 |
| --- | --- | --- | --- | --- | --- | --- | --- | --- | --- |
| Trivedi et al. 2020 | Yes | Yes | Yes | Yes | Yes | Yes | Yes | Yes | Yes |

**Key (With answers: Yes, No, Unclear or Not Applicable)**

1. Is it clear in the study what is the ‘cause’ and what is the ‘effect’ (i.e. there is no confusion about which variable comes first)?
2. Were the participants included in any comparisons similar?

3. Were the participants included in any comparisons receiving similar treatment/care, other than the exposure or intervention of interest?
4. Was there a control group?

5. Were there multiple measurements of the outcome both pre and post the intervention/exposure?

6. Was follow up complete and if not, were differences between groups in terms of their follow up adequately described and analyzed?
7. Were the outcomes of participants included in any comparisons measured in the same way?

8. Were outcomes measured in a reliable way?

9. Was appropriate statistical analysis used?
